# Lymph Node Dissection of Choice in Older Adult Patients with Gastric Cancer: A Systematic Review and Meta-Analysis

**DOI:** 10.3390/jcm13247678

**Published:** 2024-12-17

**Authors:** Camilo Ramírez-Giraldo, Violeta Avendaño-Morales, Isabella Van-Londoño, Daniela Melo-Leal, María Isabel Camargo-Areyanes, Luis Carlos Venegas-Sanabria, Juan Pablo Vargas Vargas, Edgar Javier Aguirre-Salamanca, Andrés Isaza-Restrepo

**Affiliations:** 1 Hospital Universitario Mayor-Méderi, Bogotá 111411, Colombia; violetavendano@gmail.com (V.A.-M.); luis.venegas@mederi.com.co (L.C.V.-S.); javaguirre07@gmail.com (E.J.A.-S.); andres.isaza@urosario.edu.co (A.I.-R.); 2 Universidad del Rosario, Bogotá 111221, Colombia; isabellavanl@gmail.com (I.V.-L.); danielameloleal@gmail.com (D.M.-L.); misabelcca@gmail.com (M.I.C.-A.); juanpvargas0125@gmail.com (J.P.V.V.)

**Keywords:** gastric cancer, lymphadenectomy, lymph node dissection, older adults, prognosis

## Abstract

**Background:** Although the current literature has shown an increasing interest in surgical treatment of gastric cancer (GC) in older adults in recent years, there is still no consensus on proper management in this subgroup of patients. This study was designed with the objective of evaluating the current evidence that compares limited lymph node dissection with extended lymph node dissection in older adult patients (≥65 years) coursing with resectable GC. **Methods:** A systematic review of PubMed, Cochrane library, and ScienceDirect was performed according to PRISMA guidelines. All studies before 2018 were selected using a systematic review by Mogal et al. Studies were eligible for this meta-analysis if they were randomized controlled trials or non-randomized comparative studies comparing limited lymph node dissection versus extended lymph node dissection in patients with resectable GC taken to gastrectomy. **Results:** Seventeen studies and a total of 5056 patients were included. There were not any statistically significant differences in OS (HR = 1.04, CI95% = 0.72–1.51), RFS (HR = 0.92, CI95% = 0.62–1.38), or CSS (HR = 1.24, CI95% = 0.74–2.10) between older adult patients taken to limited and extended lymphadenectomy in addition to gastrectomy as the current surgical treatment for GC. Although a higher rate of major complications was observed in the extended lymphadenectomy group, this difference was not statistically significant in incidence between both groups of patients (OR = 1.92, CI95% = 0.75–4.91). **Conclusions:** Limited lymphadenectomy must be considered as the better recommendation for surgical treatment for GC in older adult patients, considering the oncological outcomes and lower rates of complications compared with more radical lymph node dissections.

## 1. Introduction

Gastric cancer (GC) is the fifth most common malignant disease, amounting to either the second or third highest rates of mortality worldwide depending on the source [1,2]. Its incidence increases proportionally with age; according to Globocan’s predictions, GC’s crude rate for all age groups is estimated to be 12.3/100,000 people, while it goes up to 89.1/100,000 people in the population older than 70 years [3,4]. These data become even more relevant considering the fast-growing rates of people over 60 years old, prompting the United Nations to declare the decade between 2021 and 2030 as the “Decade of Healthy Aging” while urging the development of programs focused on the well-being of older individuals considering the current demographic curve change [5]. However, clinical research tends to exclude the older adult population from data analysis in GC [6]. Although GC is a public health matter, there are still multiple knowledge gaps in areas including inclusive screening strategies and timely diagnosis, the behavior of different biological variants, and customized or classified-by-age group approaches to management in older adults that could guarantee better outcomes.

The current treatment of choice for locally advanced GC is complete surgical resection (R0), with the evidence reporting better results when paired with chemotherapy [7,8]. Surgical resection on an intention-to-treat basis requires an extensive lymph node dissection, as the number of metastatic lymph nodes resected is one of the most important prognostic aspects [9,10,11]. Current international treatment guidelines recommend that the dissection of 15 or more lymph nodes be sent for histopathological studies as ideal for both proper diagnostic stratification and overall patient survival rates. Although there is still some controversy between different specialized GC centers in the East and West, nowadays, a D2 lymphadenectomy is considered the standard surgical procedure for localized resectable gastric cancer [12,13,14]. 

However, the appropriate therapeutic approach for GC in older adult patients is still a controversial subject for which there is no consensus on the extension of lymphadenectomy or chemotherapy schemes to follow. It is thus pertinent to consider this population’s remaining life span in relation to the extension of lymphadenectomy, considering the current markers for successful treatment are determined in terms of time into 3- or 5-year overall survival (OS), relapse-free survival (RFS), and cancer-specific survival (CSS). Most current studies support the D2 lymphadenectomy as the standard surgical procedure after evaluating its benefits for up to 15 years of patient follow-up [15,16], which is a significant extension of time considering older adults’ lifespan. Other important factors to take into account in this population are comorbidity and fragility as they determine a higher risk of complications, postoperative mortality, and mortality due to a different etiology than the primary tumor [4,17]. These considerations often also suggest against performing or completing chemotherapy schemes in this population, further highlighting the importance of a complete surgical approach for achieving optimistic results [18].

Although the current literature has shown an increasing interest in the surgical treatment of gastric cancer in older adults in recent years, there is still no consensus on proper management in this subgroup of patients [19,20]. Considering the aforementioned, this study was designed with the objective of evaluating the current evidence that compares limited lymph node dissection (D0, D1, or D1 plus) with extended lymph node dissection (D2, D2 plus, and D3) in older adult patients (≥65 years) coursing with resectable GC. 

## 2. Materials and Methods

A systematic review and meta-analysis was performed following the “Preferred Reporting Items for Systematic Reviews and Meta-Analysis (PRISMA)” guidelines [21,22] and “A MeaSurement Tool to Assess systematic Reviews (AMSTAR 2)” [23]. This protocol was registered on PROSPERO under number CRD42023487791.

### 2.1. Literature Search and Inclusion Criteria 

A search of the literature on Pubmed and Embase from January 2018 to December 2023 was performed using the following search terms: [“lymphadenectomy” OR “lymph node dissection” OR “D1” OR “D2”] AND [“gastric cancer” OR “gastrectomy”]. All studies before 2018 were selected using a systematic review by Mogal et al. [24]. Studies were eligible for this meta-analysis if they were randomized controlled trials or non-randomized comparative studies comparing limited lymph node dissection (D0, D1, or D1 plus) versus extended lymph node dissection (D2, D2 plus, or D3) in patients with resectable GC taken to gastrectomy and were published in English. Studies were excluded if they did not provide enough information to calculate the hazard ratio (HR) and its 95% confidence interval (CI95%) for OS or if it was not possible to obtain other results of interest for this study. If a study included overlapping cohorts, we chose to analyze the study with the largest cohort. In cases where propensity score matching was performed, data were extracted from the sample after matching. Studies were initially selected by reviewing the title and abstract, and eligible articles were then reviewed using the complete manuscript. Two authors (C.R.-G. and I.V.-L.) evaluated article eligibility independently while discrepancies were resolved using a third investigator (A.I.-R.). All references detected were saved electronically in Rayyan software (specific for systematic reviews) [25]. 

### 2.2. Data Extraction 

Two authors (C.R.-G. and I.V.-L.) independently extracted all data from eligible studies using standardized forms. Extracted data included the first author, publication year, country, follow-up time duration, study design, sample size group in limited and extended lymph node dissection groups, demographical and clinical characteristics of study participants, and results of interest to this study.

### 2.3. Risk of Bias and Quality Control

Randomized clinical trials were evaluated using the Cochrane Risk of Bias Assessment Tool v.2. (RoB2) [26]. This instrument evaluates the risk of bias in five different domains as follows: arising from the randomization process, deviations from intended interventions, missing outcome data, measurement of the outcome, and selection of the reported result. According to the abovementioned evaluation, each study was classified as having either a low risk of bias, some concerns of bias, or a high risk of bias. Nonrandomized studies’ risks of bias were assessed using the Risk of Bias in Nonrandomized Studies of Interventions (ROBINS-I) instrument [27], which takes into account the following seven domains: bias due to confounding, in the selection of participants for a study, in the classification of interventions, due to deviations from intended interventions, due to missing data, due to the measurement of the outcome measurement, and due to the selection of the reported result. According to these domains, studies were classified as having either a low, moderate, high, or critical risk of bias. Studies were deemed high quality if they were classified as having either a moderate or low risk of bias and low quality if they were at a high or critical risk of bias. Two authors (C.R.-G. and I.V.-L.) independently evaluated study quality, while any discrepancies were solved by means of a third reviewer (AI).

### 2.4. Statistical Analysis 

The primary outcomes were OS, RFS, CSS, and postoperative complication rates. HR for OS, RFS, and CSS and odds ratio (OR) for postoperative complications were used as a measure of effect to evaluate prognosis in the meta-analysis. Additionally, the HR for the age variable was used as a measure of effect in the studies that did not specifically evaluate older adult patients, with the objective of reviewing whether age is an independent risk factor for OS.

The HR and their respective CI95% values were extracted from the text directly. A random effects model based on Der Simonian and Laird’s method was employed [28]. To further illustrate the results of this meta-analysis, different forest plots were generated. Publication bias was assessed using the Eggers’ test and was graphed as funnel plots. Heterogeneity between studies was evaluated using Cochran’s Q test and/or the Higgins test (with the I^2^ statistic to measure the grade of variation non-attributable to chance alone). Heterogeneity was then classified as low (I^2^ < 25%), moderate (I^2^ = 25% to 75%), or high (I^2^ > 75%). All models were performed using random effects because of the clinical and methodological heterogeneity presented in the included studies. Sensitivity analyses were performed to determine the robustness of the results. 

To review differences between subgroups and potential causes of the observed heterogeneity, an analysis by subgroups was performed according to the type of study (randomized controlled trials versus non-randomized comparative studies) and the publication date (on or before 2010 versus after 2010). 

The statistical analysis was performed using R version 4.3.0 in the RStudio 2023.03.1 environment, with the package meta (General Package for Meta-Analysis) version 6.2-1 [29]. A *p* value of <0.05 was considered as statistically significant.

## 3. Results

### 3.1. Literature Search Results

**Figure 1:**
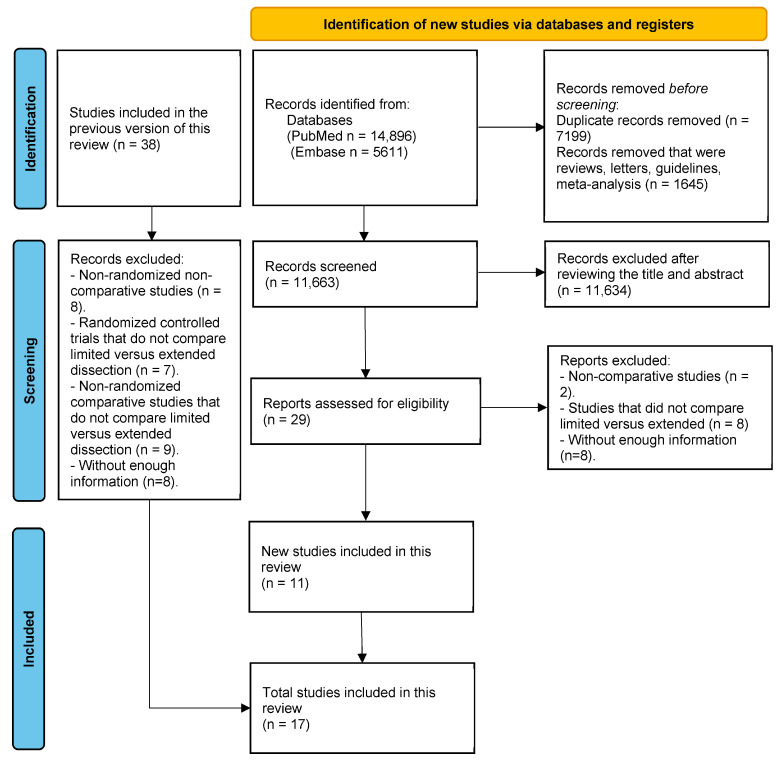
Flowchart representing information flow in each different stage of this systematic revision using PRISMA.

We identified 11,663 records after removing duplicates, reviews, letters, guidelines, and meta-analyses, followed by a screening of titles and abstract reviews, and subsequently, 29 full-text articles were reviewed. Studies were excluded if they did not compare limited versus extended lymphadenectomy according to our definition, if they were not comparative studies, and if they lacked enough data to extract for analysis [8,30,31,32,33,34,35,36,37,38,39,40,41,42,43]. Out of the studies included from the systematic revision by Mogal et al. [24], studies were excluded if they were non-randomized non-comparative, if they did not compare extended versus limited lymphadenectomy according to our definition, and if they did not have enough information to extract [6,44,45,46,47,48,49,50,51,52,53,54,55,56,57,58,59,60,61,62,63,64,65,66,67,68]. Finally, 17 studies were considered eligible for this systematic review and meta-analysis (Figure 1).

### 3.2. Description of Included Studies

The 17 included studies totaled 5056 patients distributed in the following manner: six studies that reported a total of 839 subjects included older adult patients only, out of which 444 subjects (53%) were taken to extended lymphadenectomy and 395 subjects (47%) were taken to limited lymphadenectomy (Table 1). Furthermore, 11 studies totaled 4217 patients that were not reported or defined by a specific age group, out of which 2628 subjects (62%) were taken to extended lymphadenectomy and 1589 subjects (38%) were taken to limited lymphadenectomy (Table 2). For the study by Zhang et al., only data from the D1 vs. D2 portion was included, considering that D2 is the current standard of treatment [69].

### 3.3. Quality Assessment

The overall quality of the non-randomized comparative studies comparing extended versus limited lymphadenectomy was low, mainly because of the presence of baseline confounding and selection bias according to the ROBINS-I tool. Based on the Rob2 tool, two randomized controlled trials showed a low risk of bias, and two showed some concerns for risk of bias (Supplementary File S1).

### 3.4. Overall Survival 

**Figure 2:**
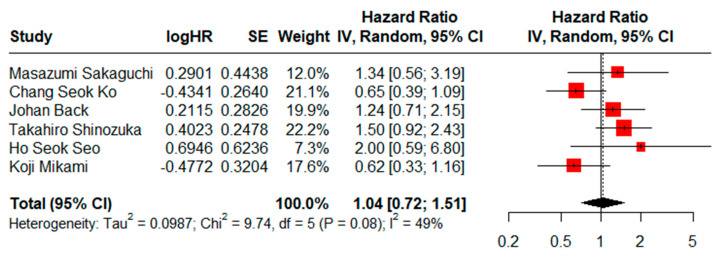
Forest plot for OS comparing limited versus extended lymphadenectomy [4,17,18,70,71,72].

HRs for OS could be assessed in six studies. There was not a statistically significant difference (HR = 1.04, CI95% = 0.72–1.51) using a random effects model and moderate heterogeneity (I^2^ = 49%, Cochran’s Q test, *p* = 0.08) (Figure 2), while the result of the sensitivity analysis proved to be stable (Supplementary File S2). No evidence of publication bias was found when generating graphic funnel plots (Supplementary File S3). There also was no evidence of publication bias using the Eggers test (*p* = 0.6290).

### 3.5. Relapse-Free Survival

**Figure 3:**
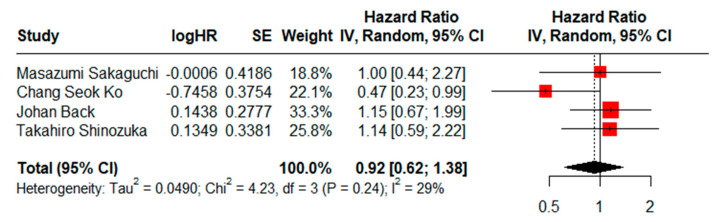
Forest plot for RFS comparing limited versus extended lymphadenectomy [4,17,18,70].

HRs for RFS could be assessed in four studies. There was not a statistically significant difference (HR = 0.92, CI95% = 0.62–1.38) using a random effects model and moderate heterogeneity (I^2^ = 29%, Cochran’s Q test, *p* = 0.24) (Figure 3), and the result of the sensitivity analysis proved to be stable (Supplementary File S2). No evidence of publication bias was found when generating visual funnel plots (Supplementary File S3). There was also no evidence of publication bias using the Eggers test (*p* = 0.5014).

### 3.6. Cancer-Specific Survival

**Figure 4:**
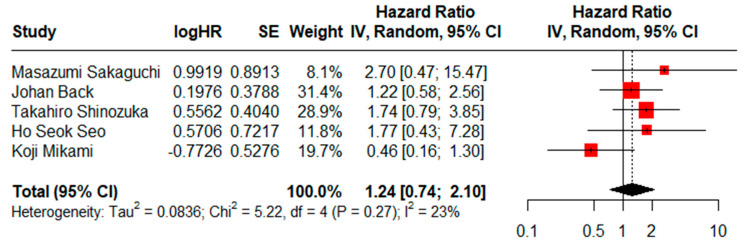
Forest plot for CSS comparing limited versus extended lymphadenectomy [4,17,70,71,72].

HRs for CSS could be assessed in five studies. There was not a statistically significant difference (HR = 1.24, CI95% = 0.74–2.10) using a random effects model and moderate heterogeneity (I^2^ = 23%, Cochran’s Q test, *p* = 0.27) (Figure 4), and the result of the sensitivity analysis proved to be stable (Supplementary File S2). No evidence of publication bias was found when generating graphic funnel plots (Supplementary File S3). There was also no evidence of publication bias using the Eggers test (*p* = 0.7893). 

### 3.7. Postoperative Complications (Clavien–Dindo ≥ 3)

**Figure 5:**
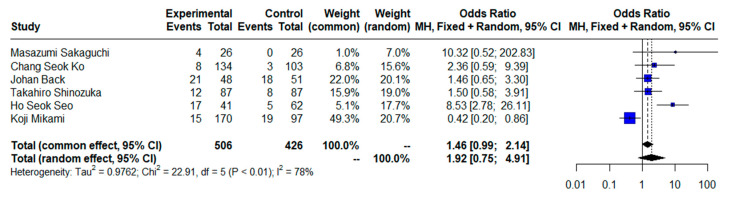
Forest plot for complications with Clavien–Dindo ≥ 3 comparing limited versus extended lymphadenectomy [4,17,18,70,71,72].

ORs for postoperative complications (Clavien–Dindo ≥ 3) could be assessed in six studies. There was not a statistically significant difference (OR = 1.92, CI95% = 0.75–4.91) using a random effects model and moderate heterogeneity (I^2^ = 78%, Cochran’s Q test, *p* < 0.01) (Figure 5), while the result of the sensitivity analysis identified a statistically significant difference in ORs for complications between groups when excluding the study by Mikami et al. [72] (OR = 2.67, CI95% = 1.21–5.87) using a random effects model and moderate heterogeneity (I^2^ = 51.1%, Cochran’s Q test, *p* = 0.08) (Supplementary File S2). No evidence of publication bias was found when generating graphic funnel plots (Supplementary File S3). There was also no evidence of publication bias using the Eggers test (*p* = 0.1705).

### 3.8. Age as an Independent Risk Factor for OS

**Figure 6:**
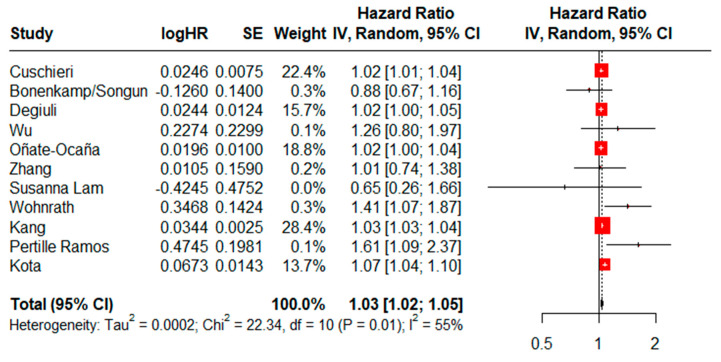
Forest plot for age as a risk factor for OS [69,73,76,77,81,82,83,84,85,86].

HRs for age as an independent risk factor for OS could be assessed in eleven studies. There was a statistically significant difference (HR = 1.03, CI95% = 1.02–1.05) using a random effects model and moderate heterogeneity (I^2^ = 55%, Cochran’s Q test, *p* = 0.01) (Figure 6), while the result of the sensitivity analysis proved to be stable (Supplementary File S2). No evidence of publication bias was found when generating graphic funnel plots (Supplementary File S3), while there was no evidence of publication bias using the Eggers test (*p* = 0.5686). In the analysis by subgroups according to study design and year of publication, a statistically significant difference between both subgroups was maintained (Supplementary File S4).

## 4. Discussion 

In this current study, 11 manuscripts were deemed eligible for analysis, with an additional 6 manuscripts that were previously identified and reviewed by Mogal et al. [24], for a total of 5056 patients. Moreover, it is important to consider that many of the available clinical studies in the current literature include an age limit and/or adequate physical condition with no serious comorbidities cutoff that precludes safe D2 dissection, which greatly limits the inclusion of older adult patients for analysis [15,16,74,79].

Considering available evidence in the scientific literature, there were not any statistically significant differences in OS, RFS, or CSS between older adult patients taken to limited versus extended lymphadenectomy in addition to gastrectomy as the current surgical treatment for GC. Although a higher rate of major complications (defined as a Clavien–Dindo score ≥3) was observed in the extended lymphadenectomy group, this difference was not statistically significant in incidence between both groups of patients. When excluding the study by Mikami et al. [72] from the sensitivity analysis, the extended lymphadenectomy group of patients presented a statistically significant higher risk of complications (OR = 2.67, CI95% = 1.21–5.87).

Performing limited lymphadenectomy can be considered the optimal choice when faced with older adult patients, considering that no significant differences between results (OS, RFS, and CSS) were identified in the treatment for GC in older adult patients regarding lymphadenectomy extension while summing all special considerations to be thought of in this group. As mentioned in the Introduction, the current evidence that supports extended lymphadenectomy as the procedure that yields better results for OS in patients with GC comes from Dutch and Italian studies that proved their benefits after a 15-year follow-up period. However, these long periods are irrelevant when faced with advanced ages considering the average lifespan [15,16]. Many studies on GC exclude this group of patients (older adults) because survival cannot be adequately studied in this age group [6].

As lymphadenectomy extension in this age group does not depend on oncological results (and are nonetheless similar), associated morbimortality to the procedure acquires a higher relevance considering that older adults suffer from more comorbidities, immunological decline, lengthier healing periods, clotting disorders, fragility, and lower tolerance to surgical stress [17]. Although this meta-analysis did not find a statistically significant difference in this variable, a significatively higher risk of complications were identified in patients taken to extended lymphadenectomy when the study by Mikami et al. was excluded from the sensitivity analysis [72]. Their study was also the only one out of the five studies included that evidenced a trend towards more complications in the limited lymphadenectomy group. The study analyzed a retrospective series of patients on which the decision to perform limited lymphadenectomy was made according to surgeon criteria, with the intention to lower complication rates in patients with major comorbidities or a deficient nutritional or cardiopulmonary state, or based on intraoperative findings. 

In other patient series, findings regarding morbidity for lymphadenectomy were contradictory. Rausei et al. evidenced that patients over 70 years of age taken to extended lymphadenectomy presented more complications than patients under 70 years of age, with a significant difference in OS [87]. Ji Hoon Kang et al. reported that lymphadenectomy extension was not a risk factor for major complications [84]. This is in line with a population-based cohort study by Brenkman et al., where it was evidenced that in patients over 75 years of age, overall survival increased proportionately with the amount of lymph nodes dissected, without any significant increases in the appearance of complications [56]. On the other hand, Shinozuka et al. [17], Sakaguchi et al. [4], Seok Seo et al. [71], and Passot et al. [59] concluded that D2 lymphadenectomy in older adult patients provides fewer benefits to survival compared with the higher rates of complications. Taking our own clinical and surgical experience into account alongside the quality of the studies evaluated and the different selection biases identified in all series analyzed, we arrived at the conclusion that extended lymphadenectomy is associated with a higher risk of presenting postoperative complications. Therefore, limited lymphadenectomy could reduce postoperative complication rates; this is in line with the conclusions by Takama et al. [88] and Zhang et al. [6]. Some other studies also suggested that the appearance of postoperative complications in older adult patients [84,85,86,87] can be associated to worse oncological outcomes [17]. However, this relationship was not evident in a meta-analysis by Onagi et al. [89]. 

Moreover, therapeutical outcomes for older adult patients differ from those for other age groups as quality of life, independence, and patient autonomy are preferred over lengthening the patient’s lifespan [90,91,92,93]. This becomes even more relevant if we consider the results of an older adult cohort of patients with early GC in Japan, where it was evidenced that more than half of older adult patients could die from reasons other than gastric cancer [18,94]. Another important fact to highlight is that most studies did not evaluate older adult patients based on a comprehensive geriatric assessment [95,96], and when analyzing observational studies included in this study (which were mostly retrospective cohorts), most surgeons were inclined towards limited lymphadenectomy in fragile, comorbid, higher-risk patients [70,72,87]. Additionally, lymphadenectomy extension is irrelevant when there are compromised lymph nodes or if it has evolved into a systemic disease [32]. Nevertheless, it is also true that older adult patients were less likely to undergo perioperative adjuvant chemoradiation therapy despite studies affirming its survival benefit [97]. In order to create more specific recommendations for this age group, we must have better-organized data, either by decades of life or following standardized geriatric assessments, considering that this age group’s characteristics vary widely and change quite rapidly, as current studies tend to group all patients as older adults regardless of if they are between the ages of 65 and 90 or even higher. 

The link between aging and cancer is complex. Although there is clear evidence that cells entering a senescent state can act as a barrier to tumorigenesis, some studies have demonstrated that in certain conditions, persistent senescent cells can acquire pro-tumorigenic properties; thus, senescence has dual roles in tumorigenesis [98]. Therefore, GC in older adult patients tends to follow a less aggressive biological and clinical course. This could be explained by the higher rates of intestinal-type histological patterns, its better differentiation and distal location in the stomach, which favors the decision to perform less radical procedures [87]. 

Age was identified as an independent risk factor for lower OS in patients taken to gastrectomy under GC indication (HR = 1.03, CI95% 1.02–1.05). This implies that age is a risk factor regardless of disease staging, histological pattern, type of procedure, and tumor location, among others, and as such, older adult patients must be carefully selected for surgery. Moreover, as the average lifespan has positively increased in the general population, so has the age of presentation for patients with GC. This phenomenon was evidenced in South Korea, where the incidence of GC in patients over 70 years old for GC increased from 9.1% in 1995 to 25% in 2014. Thus, aging has come into prominence as a critical issue to be considered in surgery for GC [18].

Some of the limitations of this study are related to the fact that results from different studies are difficult to compare for various reasons. First, the cut-off for the definition of a patient as an “older adult” is not homogenous, and comorbidities were calculated using several different scoring systems. Furthermore, as mentioned above, a better analysis is lacking as it is not possible to stratify different groups of patients from a geriatric standpoint using a standardized geriatric assessment as a reference point or by decade subgroups, considering the widely varying characteristics found depending on a specific age [99]. Moreover, considering that surgery is only a portion of the overall current treatment guidelines for GC, another limitation of this study constitutes the lack of an analysis of the role of chemotherapy in GC survival. This is in line with other studies in which the administration of different regimens with different schedules, the frequent exclusion of older adult high-risk patients from oncological trials, the lack of offers, and the contraindication for chemotherapy in this age group perpetuate the difficulty of analyzing this variable in this population. Finally, the quality of the observational studies included was low, mainly because of selection bias [19]. 

This study thus concludes that limited lymphadenectomy must be considered as the better recommendation for surgical treatment for GC in older adult patients, considering the oncological outcomes and lower rates of complications compared with more radical lymph node dissections. Nevertheless, it is important to individualize these decisions considering not only chronological but biological age [100] as well to establish the best management conduct possible. More clinical studies of better quality focused on older adult patients are needed, in which a comprehensive geriatric assessment is performed to generate better recommendations based on higher-quality evidence.

## Figures and Tables

**Table 1:** Studies that compared limited and extended lymphadenectomy in older adults.

Author/Study Group/Number of Patients	Study Period	Median/Mean * Aged Comparing Limited Versus Extended	Median/Mean * Nodes Removed Comparing Limited Versus Extended	Overall Survival Comparing Limited Versus Extended	Operative Morbimortality Comparing Limited Versus Extended	Comments
Sakaguchi et al. [4] N = 52	2011–2017	L: 80 E: 81	L: 38 E: 44	3-year OS L: 68.8% E: 68.8%	Clavien–Dindo ≥3 L: 0% E: 3.9%	Patients ≥75 years. Laparoscopic gastrectomy. There were no statistically significant differences in the retrieved lymph nodes between extended versus limited lymphadenectomy. There were no statistically significant differences in the OS.
Ko et al. [18] N = 144	2007–2015	L: 78.61 * E: 78.82 *	L: 25.04 * E: 34.19 *	3 year OS HR: 1.72 5-year OS HR: 1.54	Clavien–Dindo ≥3 L: 2.8% E: 8.3%	Patients ≥75 years. Adjuvant chemotherapy was 36.1% in the limited group and 27.8% in the extended group. There were no significant differences in Clavien–Dindo ≥3 scores between groups (*p* = 0.289). There were statistically significant differences in the 5-year RFS (HR 2.11 (95%CI 1.01–4.40)). There were no statistically significant differences in the 5-year OS (HR 1.54 (95%CI 0.92–2.59)).
Back et al. [70] N = 99	2000–2015	NR	L: 12.0 E: 20.0	OS L: 32.7 months E: 31.7 months	Clavien–Dindo ≥3 L: 17.6% E: 20.8%	Patients ≥75 years. Patients in the D1 group were older, had higher ASA class, and had more frequently cerebrovascular disease and anticoagulation medication than patients in the D2 group. The median follow-up time was 32.5 months. Morbidity and mortality were similar in both groups. The D1 and D2 groups were similar in OS, CSS, and RFS.
Shinozuka et al. [17] N = 174	2010–2014	L: 83.0 E: 82.0	L: 24.0 E: 32.0	3-year OS L: 77.6% E: 72.8%	Clavien–Dindo ≥3 L: 8.0% E: 11.5%	Patients ≥80 years. The patients in the D2 group had a significantly longer median operation time and more blood loss compared with those in the non-D2 group. There was no significant difference between the two groups in grade III or greater postoperative complications. No significant differences in CSS and RFS were found between the groups (HR 1.70; *p* = 0.180 and HR 1.14; *p* = 0.687, respectively). There were no significant differences in OS between the groups (HR 1.49, *p* = 0.104).
Seo et al. [71] N = 103	2006–2016	L: 82.8 * E: 82.8 *	L: 35.1 * E: 43.8 *	NR	Clavien–Dindo ≥3 L: 4.8 E: 17.1	Patients ≥80 years. The mean follow-up period for the 103 enrolled patients was 33.1 ± 23.4 months. No differences in Clavien–Dindo classification or duration until discharge were observed between groups. No differences in OS rates according to the extent of lymphadenectomy were detected between groups (*p* = 0.223). Cox regression analysis: History of coronary artery disease, Clavien–Dindo ≥ 3, and TNM stage were independent risk factors for OS. Logistic regression analysis: D2 increased the risk for Clavin–Dindo ≥ 3.
Mikami et al. [72] N = 267	2001–2011	L: 78.3* E: 75.2*	L: 27.3 E: 36.1	5-year OS L: 52.6% E: 82.4%	Clavien–Dindo ≥3 L: 18.6 E: 15.3	Patients ≥70 years. Type of lymphadenectomy was determined preoperatively by the surgeon considering cardiopulmonary function, other comorbidities, and other patient conditions or based on intraoperative findings. Morbidity was similar between groups (*p* = 0.489). The 5-year OS rate was significantly lower in the limited group than the extended group (52.6% vs. 82.4%, *p* < 0.001). Among patients with Stage II and Stage III disease, 5- year OS rates of the limited group were significantly lower than in the extended group (41.3% vs. 72.3%, *p* = 0.001). Multivariate analysis showed that age, gender, body mass index, prognostic nutrition index, and tumor stage were independent prognostic factors for poor overall survival.

L: limited; E: extended; IQR: interquartile range; CI: confidence interval; OS: overall survival; NR: reported; HR: hazard ratio; RFS: relapse-Free Survival; CSS: cancer-Specific Survival.

**Table 2:** Studies that compared limited and extended lymphadenectomy (age group non-defined).

Author/Study Group/Number of Patients	Study Period	Median/Mean * Aged Comparing Limited Versus Extended (Years)	Median/Mean * Nodes Removed Comparing Limited Versus Extended	Overall Survival Comparing Limited Versus Extended	Operative Morbimortality Comparing Limited Versus Extended	Comments
MRC ST01 [73,74] N = 400	1987–1994	L: 67.0 E: 67.0	L: 13.0 E: 17.0	5-year OS L: 35% E: 33%	Minor/serious/fatal complications L: 28% E: 46%	Patients were excluded if they had serious co-morbid cardiorespiratory disease that would preclude a safe D2 resection. Non-pancreas/spleen-preserving D2 dissection. Older patients, males, and stage II or III patients all experienced poor survival rates. D2 resection offered no significant survival benefit over D surgery.
Dutch gastric cancer trial [16,75,76] N = 711	1989–1993	L: 67.0 E: 65.0	L: 18.4 E: 31.5	5-year OS L: 45% E: 47% 15-year OS L: 21% E: 29%	Mortality L: 4% E: 10% Morbidity L: 25% E: 43% Reoperation L: 8% E: 18%	Inclusion criteria were age younger than 85 years and an adequate physical condition. Non-pancreas/spleen-preserving D2 dissection. Significant risk factors for postoperative mortality in the logistic regression analysis were age >70 versus ≤70 years (OR 3.55, 95% CI 1.91–6.61, *p* < 0.0001), D2 versus D1 lymphadenectomy (OR 2.64, 1.38–5.04, *p* = 0.003). Improved OS for D2 versus D1 in TNM stage II patients (33% vs. 15%), N2 disease (19% vs. 0%).
Italian gastric cancer study group [15,77,78] N = 267	1998–2006	L: 64.0 E: 62.0	L: 25.0 E: 33.0	5-year OS L: 66.5% E: 64.2% 15-year OS L: 51.3% E: 46.8%	Mortality L: 3.0% E: 2.2% Morbidity L: 12% E: 17.9%	Patients less than 80 years that were in an adequate physical condition with no serious co-morbid cardiorespiratory or renal disease precluding safe D2 dissection. Spleen- and pancreas-preserving D2 dissections. There was no significant difference between groups regarding morbidity. A significantly worse survival was observed in patients aged 70 years or more (*p* = 0.033). Univariable analysis of the 5-year CSS rate showed a survival benefit for patients aged 70 years or more who had a D1 gastrectomy (75% versus 51% for D2 resection; *p* = 0.018). For pT > 1 and N+ tumors, improved 5-year OS (51% and 35%) and CSS (59% and 38%) for D2 versus D1. Fifteen-year OS and CSS were better after D1 in patients older than 70 years (*p* = 0.003 and *p* = 0.006). Fifteen-year CSS was significantly higher after D2 in pT > 1 and N+ tumors (29.4% vs. 51.4%, *p* = 0.035).
Wu et al. [79,80] N = 221	1993–1999	L: 63.0 E: 65.2	L: 19.4 E: 37.2	5-year OS L: 53.6% E: 59.5%	Mortality L: 0% E: 0% Morbidity L: 7.3% E: 17.1%	Inclusion criteria were age less than 75 years and a physical condition suitable for either operation. Multivariable analysis: Age >65 versus ≤65 years (HR 1.25, 95% CI 0.80–1.97, *p* = 0.326) Multivariate analyses showed that nodal disease, a tumor in the whole stomach, Bormann type III and IV appearances, and allocation to D1 surgery were associated with poor survival.
Oñate-Ocaña et al. [81] N = 219	1987–1998	L: 55.2 * E: 57.5 *	L: 16.8 * E: 23.6 *	5-year OS L: 35% E: 64%	Mortality L: 6.8% E: 8.6% Morbidity L: 16.9% E: 19.5%	Univariate analysis of prognostic factors: Age risk ratio 1.02 (95% CI = 1.0003–1.04) (*p* = 0.046). Multivariate analysis using the Cox model showed that the most important prognostic determinants were T stage, N stage, the serum albumin level, total gastrectomy, and extended lymphadenectomy.
Zhang et al. [69] N = 376	1980–2005	L: 55.0 E: 55.0	L: 21.0 * E: 23.0 *	5-year OS L: 37.4% E: NR	Mortality L: 1.6% E: 0.5% Morbidity L: 14% E: 13%	Inclusion criteria were age younger than 76 years and an adequate physical condition. No significant difference in OS between the D1 and D2 groups. The multivariate analyses showed that >7 cm in tumor size, the upper third tumor and the whole stomach tumor, Bormann III type, N3 disease, and D1 and D2 dissection were significantly associated with poor survival.
Susanna Lam et al. [82] N = 78	1996–2016	L: 67.4 * E: 64.9 *	L: 15.0 * E: 20.3 *	5-year OS L: 52.1% E: 76.2% 10-year OS L: 52.1% E: 76.2%	Mortality L: 2% E: 0% Clavien–Dindo ≥3 L: 12.82% E: 5.12%	The prognostic factor for patients undergoing gastrectomy with D1/D1+ or D2 lymphadenectomy was age ≥70 versus <70 years (HR 1.66, 95% CI 0.714–3.881, *p* = 0.237). Advanced age (≥70 years) was associated with postoperative complications (*p* = 0.006); however, these were mostly Clavien–Dindo grade I and II complications. Older age was not associated with 30-day mortality. OS was significantly longer in patients in the D2 group (*p* = 0.008). Multiple Cox analysis showed that advanced tumor stage (stages III and IV), lymphadenectomy type (D1), and the presence of postoperative complications were independent predictors of poor OS.
Wohnrath et al. [83] N = 656	1994–2015	L: 65.0 E: 63.0	L: 20.0 E: 28.0	5-year OS L: 43.2% E: 26.0% 10-year OS L: 9.4% E: 30.6%	Mortality L: 5.9% E: 4% Clavien–Dindo ≥3 L: 11.9% E: 8.8%	According to multivariate analysis, the risk of death increased with older age (≥70-years), high-grade tumors, lesions ≥ 5 cm, positive nodes ≥ 3, and extra-gastric resections. D2 lymphadenectomy improved the median OS (37 versus 16 months) and 3-years (51.1 versus 32.2%), 5-years (43.2 versus 26), and 10 years OS (30.6 versus 9.4%).
Ji Hoon Kang et al. [84] N = 370	2008–2014	L: 62.5 * E: 62.9 *	L: 40.0 * E: 45.0 *	5-year OS L: 82.3% E: 82.4%	Mortality L: 0% E: 0.9% Clavien–Dindo ≥3 L: 3.8% E: 4.3%	Pathologic stage of ≥ pT2 or pN +. D1+ versus D2 lymphadenectomy. The D2 and D1 groups showed no significant difference in OS (*p* = 0.488) or RFS (*p* = 0.705). Extension of lymphadenectomy was not an independent predictive factor for postoperative morbidity. Age, sex, BMI, pT stage, and pN stage were independent prognostic factors.
Pertille Ramos et al. [85] N = 460	2009–2017	L: 72.4 * E: 61.2 *	L: 27.0 * E: 41.3 *	OS L: 61.6% E: 76.0%	Mortality L: 17.8% E: 3.4% Clavien–Dindo ≥3 L: 24.7% E: 12.4%	D1 lymphadenectomy was chosen in unfavorable clinical conditions. The median follow-up time was 24.4 months. In the D1 group, 14 patients were older than 70 years; 10 had CCI ≥5 and 10 were ASAIII/IV. Major postoperative complications (Clavien–Dindo ≥ III) were significantly higher in the D1 group (24.7% vs. 12.4%, *p* < 0.001) Multivariate analysis showed a statistically significant impact on survival of age ≥70 years, CCI ≥5, total gastrectomy, D1 lymphadenectomy and advanced stages (III/IV).
Kota et al. [86] N = 466	2002–2014	L: 69.5 E: 65.5	L: 32.0 E: 40.0	5-year OS L: 78.2% E: 85.1%	Mortality L: 0% E: 0.2% Clavien–Dindo ≥2 L: 10% E: 26.8%	Patients with cT1N1, cT2N0-1, or cT3N0. D1+ versus D2 lymphadenectomy. The number of patients with higher age and a higher comorbidity index was greater in the D1+ group than in the D2 group. Postoperative complications were significantly lower in the D1+ group than in the D2 group (10.0% vs. 26.8%, *p* = 0.004). No statistically significant difference in 5-year OS (*p* = 0.146) or CSS (*p* = 0.807) between groups was noted. Multivariable analysis for overall survival identified age, clinical node-positive status, high CCI (≥3), advanced pathological stage (≥III), and the presence of postoperative complications (grade ≥II) as independent prognostic factors.

L: limited; E: extended; NR: reported; CI: confidence interval; HR: hazard ratio; OR: odds ratio; CCI: Charlson comorbidity index; RFS: relapse-Free Survival; CSS: cancer-Specific Survival; OS: overall survival.

## Data Availability

Data are contained within the article and supplementary materials.

## Supplementary Materials

The following supporting information can be downloaded at https://www.mdpi.com/article/10.3390/jcm13247678/s1: File S1. Quality assessment of included studies. File S2. Sensitivity analysis. File S3. Funnel plot. File S4. Subgroup analysis.

## References

[1] Bray F., Ferlay J., Soerjomataram I., Siegel R.L., Torre L.A., Jemal A. (2018). Global cancer statistics 2018: GLOBOCAN estimates of incidence and mortality worldwide for 36 cancers in 185 countries. CA Cancer J. Clin..

[2] Kelley J.R., Duggan J.M. (2003). Gastric cancer epidemiology and risk factors. J. Clin. Epidemiol..

[3] Bray F., Colombet M., Aitken J., Bardot A., Eser S., Galceran J., Hagenimana M., Matsuda T., Mery L., Piñeros M., Bray F., Colombet M., Aitken J., Bardot A., Eser S., Galceran J., Hagenimana M., Matsuda T., Mery L., Piñeros M. (2023). Cancer Incidence in Five Continents.

[4] Sakaguchi M., Hosogi H., Kanaya S. (2022). Is D2 laparoscopic gastrectomy essential for elderly patients with advanced gastric cancer? A propensity score matched analysis. J. Gastrointest. Oncol..

[5] Lozada-martinez I.D., Sebastian J., Castelblanco-toro S.M., Sarmiento M., Mazenett-granados E.A., Fredy J., Anaya M. (2024). Demographics and clinical characteristics of a new population of centenarians in Colombia. The COOLCEN cohort. Arch. Gerontol. Geriatr. Plus..

[6] Zhang C.D., Zong L., Ning F.L., Zeng X.T., Dai D.Q. (2018). Modified vs. Standard D2 lymphadenectomy in distal subtotal gastrectomy for locally advanced gastric cancer patients under 70 years of age. Oncol. Lett..

[7] Cunningham D., Allum W.H., Stenning S.P., Thompson J.N., Van de Velde C.J.H., Nicolson M., Scarffe J.H., Lofts F.J., Falk S.J., Iveson T.J. (2006). Perioperative Chemotherapy versus Surgery Alone for Resectable Gastroesophageal Cancer. N. Engl. J. Med..

[8] Kung C.H., Tsai J.A., Lundell L., Johansson J., Nilsson M., Lindblad M. (2020). Nationwide study of the impact of D2 lymphadenectomy on survival after gastric cancer surgery. BJS Open.

[9] Zhang Y.X., Yang K. (2020). Significance of nodal dissection and nodal positivity in gastric cancer. Transl. Gastroenterol. Hepatol..

[10] Marchet A., Mocellin S., Ambrosi A., Morgagni P., Garcea D., Marrelli D., Roviello F., De Manzoni G., Minicozzi A., Natalini G. (2007). The ratio between metastatic and examined lymph nodes (N ratio) is an independent prognostic factor in gastric cancer regardless of the type of lymphadenectomy: Results from an Italian multicentric study in 1853 patients. Ann. Surg..

[11] Marrelli D., Piccioni S.A., Carbone L., Petrioli R., Costantini M., Malagnino V., Bagnacci G., Rizzoli G., Calomino N., Piagnerelli R. (2024). Posterior and Para-Aortic (D2plus) Lymphadenectomy after Neoadjuvant/Conversion Therapy for Locally Advanced/Oligometastatic Gastric Cancer. Cancers.

[12] Japanese Gastric Cancer Association (2023). Japanese Gastric Cancer Treatment Guidelines 2021 (6th edition). Gastric Cancer.

[13] Lordick F., Carneiro F., Cascinu S., Fleitas T., Haustermans K., Piessen G., Vogel A., Smyth E.C. (2022). Gastric cancer: ESMO Clinical Practice Guideline for diagnosis, treatment and follow-up. Ann. Oncol..

[14] Ajani J.A., D’Amico T.A., Bentrem D.J., Chao J., Cooke D., Corvera C., Das P., Enzinger P.C., Enzler T., Fanta P. (2022). Gastric Cancer, Version 2.2022. JNCCN J. Natl. Compr. Cancer Netw..

[15] Degiuli M., Reddavid R., Tomatis M., Ponti A., Morino M., Sasako M., Rebecchi F., Garino M., Vigano L., Scaglione D. (2021). D2 dissection improves disease-specific survival in advanced gastric cancer patients: 15-year follow-up results of the Italian Gastric Cancer Study Group D1 versus D2 randomised controlled trial. Eur. J. Cancer.

[16] Songun I., Putter H., Kranenbarg E.M.K., Sasako M., van de Velde C.J.H. (2010). Surgical treatment of gastric cancer: 15-year follow-up results of the randomised nationwide Dutch D1D2 trial. Lancet Oncol..

[17] Shinozuka T., Kanda M., Ito S., Mochizuki Y., Teramoto H., Ishigure K., Murai T., Asada T., Ishiyama A., Matsushita H. (2020). D2 lymph node dissection confers little benefit on the overall survival of older patients with resectable gastric cancer: A propensity score-matching analysis of a multi-institutional dataset. Surg. Today.

[18] Ko C.S., Jheong J.H., Jeong S.A., Kim B.S., Yook J.H., Yoo M.W., Kim B.S., Lee I.S., Kim S., Gong C.S. (2022). Comparison of Standard D2 and Limited Lymph Node Dissection in Elderly Patients with Advanced Gastric Cancer. Ann. Surg. Oncol..

[19] Ruspi L., Galli F., Pappalardo V., Inversini D., Martignoni F., Boni L., Dionigi G., Rausei S. (2017). Lymphadenectomy in elderly/high risk patients: Should it be different?. Transl. Gastroenterol. Hepatol..

[20] Argillander T.E., Festen S., van der Zaag-Loonen H.J., de Graeff P., van der Zaag E.S., van Leeuwen B.L., Nagengast W.B., Verhage R.J.J., Ruurda J.P., van Munster B.C. (2022). Outcomes of surgical treatment of non-metastatic gastric cancer in patients aged 70 and older: A systematic review and meta-analysis. Eur. J. Surg. Oncol..

[21] Page M.J., McKenzie J.E., Bossuyt P.M., Boutron I., Hoffmann T.C., Mulrow C.D., Shamseer L., Tetzlaff J.M., Akl E.A., Brennan S.E. (2021). The PRISMA 2020 statement: An updated guideline for reporting systematic reviews. BMJ.

[22] Muka T., Glisic M., Milic J., Verhoog S., Bohlius J., Bramer W., Chowdhury R., Franco O.H. (2020). A 24-step guide on how to design, conduct, and successfully publish a systematic review and meta-analysis in medical research. Eur. J. Epidemiol..

[23] Shea B.J., Reeves B.C., Wells G., Thuku M., Hamel C., Moran J., Moher D., Tugwell P., Welch V., Kristjansson E. (2017). AMSTAR 2: A critical appraisal tool for systematic reviews that include randomised or non-randomised studies of healthcare interventions, or both. BMJ.

[24] Mogal H., Fields R., Maithel S.K., Votanopoulos K. (2019). In Patients with Localized and Resectable Gastric Cancer, what is the Optimal Extent of Lymph Node Dissection—D1 Versus D2 Versus D3?. Ann. Surg. Oncol..

[25] Ouzzani M., Hammady H., Fedorowicz Z., Elmagarmid A. (2016). Rayyan-a web and mobile app for systematic reviews. Syst. Rev..

[26] Sterne J.A.C., Savović J., Page M.J., Elbers R.G., Blencowe N.S., Boutron I., Cates C.J., Cheng H.Y., Corbett M.S., Eldridge S.M. (2019). RoB 2: A revised tool for assessing risk of bias in randomised trials. BMJ.

[27] Sterne J.A., Hernán M.A., Reeves B.C., Savović J., Berkman N.D., Viswanathan M., Henry D., Altman D.G., Ansari M.T., Boutron I. (2016). ROBINS-I: A tool for assessing risk of bias in non-randomised studies of interventions. BMJ.

[28] DerSimonian R., Laird N. (2015). Meta-analysis in clinical trials revisited. Contemp. Clin. Trials..

[29] Schwarzer G., Carpenter J.R., Rücker G. (2023). Meta-Analysis with R. First.

[30] Luo X., Zhou M.X., Tian W., Zeng M., Xia J.L., Zhao G.P., Hu H.L., Hao X.B., Han L.F., Liu H. (2020). A retrospective study comparing D1 limited lymph node dissection and D2 extended lymph node dissection for N3 gastric cancer. Transl. Cancer Res..

[31] Yu P., Du Y., Xu Z., Huang L., Cheng X. (2019). Comparison of D2 and D2 plus radical surgery for advanced distal gastric cancer: A randomized controlled study. World J. Surg. Oncol..

[32] Özmen M.M., Zülfikaroğlu B., Özmen F., Moran M., Özalp N., Seçkin S. (2021). D2 vs. D2 plus para-aortic lymph node dissection for advanced gastric cancer. Turkish J. Surg..

[33] Silva F.D.A., Pereira M.A., Ramos M.F.K.P., Ribeiro-Junior U., Zilberstein B., Cecconello I., Dias A.R. (2020). Gastrectomy in octogenarians with gastric cancer: Is it feasible?. Arq. Bras. Cir. Dig..

[34] Gosselin-Tardif A., Lie J., Nicolau I., Molina J.C., Cools-Lartigue J., Feldman L., Spicer J., Mueller C., Ferri L. (2018). Gastrectomy with extended lymphadenectomy: A North American perspective. J. Gastrointest. Surg..

[35] Morkavuk Ş.B., Çulcu S., Tez M., Ünal A.E. (2021). The efficiency of D1^+^ lymphadenectomy in signet ring cell carcinoma: Comparison of postoperative early and late outcomes between standard lymphadenectomy and D1^+^ lymphadenectomy. Libyan J. Med..

[36] Lorenzon L., Giudicissi R., Scatizzi M., Balducci G., Cantafio S., Biondi A., Persiani R., Mercantini P., D’Ugo D. (2020). D1-plus vs. D2 nodal dissection in gastric cancer: A propensity score matched comparison and review of published literature. BMC Surg..

[37] Tudor S., Dumitrascu T., Manuc M., Trandafir B., Ionescu M., Popescu I., Herlea V., Vasilescu C. (2018). D2 lymphadenectomy for gastric adenocarcinoma: Long-term results and the impact of surgeon experience on the survival rates. Chirurgia.

[38] Park S.H., Son T., Seo W.J., Lee J.H., Choi Y.Y., Kim H.I., Cheong J.H., Noh S.H., Hyung W.J. (2019). Prognostic impact of extended lymph node dissection versus limited lymph node dissection on pn0 proximal advanced gastric cancer: A propensity score matching analysis. J. Gastric Cancer..

[39] Elmessiry M.M., El-Fayoumi T.A., Fayed H.M., Gebaly A.A., Mohamed E.A.E. (2022). Operative and Oncological Outcomes After D2 Versus D1 Gastrectomy of Operable Gastric Cancer: An Observational Study. J. Gastrointest. Cancer..

[40] Uslu A., Zengel B., Ilhan E., Aykas A., Şimşek C., Üreyen O., Duran A., Okut G. (2018). Survival outcomes after D1 and D2 lymphadenectomy with R0 resection in stage II-III gastric cancer: Longitudinal follow-up in a single center. Turkish J. Surg..

[41] Yamagata Y., Yoshikawa T., Ishizu K., Tsutsui M., Wada T., Hayashi T. (2023). Impact of D2 Gastrectomy for Locally Advanced Gastric Cancer in the Era of Effective Adjuvant Chemotherapy. World J. Surg..

[42] Oh Y.J., Kim D.H., Eom B.W., Yoon H.M., Kim Y.W., Ryu K.W. (2021). Is Splenic Hilar Lymph Node Dissection Without Splenectomy Essential for Proximal Advanced Gastric Cancer?. Ann. Surg. Oncol..

[43] Liang Y., Cui J., Cai Y., Liu L., Zhou J., Li Q., Wu J., He D. (2019). D2 plus lymphadenectomy is associated with improved survival in distal gastric cancer with clinical serosa invasion: A propensity score analysis. Sci. Rep..

[44] Volpe C.M., Koo J., Miloro S.M., Driscoll D.L., Nava H., Douglass H. (1995). The effect of extend pymphadenectomy on survival in patients with gastric adenocarcinoma. J. Am. Coll. Surg..

[45] Kaibara N., Sumi K., Yonekawa M., Ohta M., Makino M., Kimura O., Nishidoi H., Koga S. (1990). Does extensive dissection of lymph nodes improve the results of surgical treatment of gastric cancer?. Am. J. Surg..

[46] Bostanci E.B., Yol S., Kayaalp C., Ozogul Y., Aydin C., Atalay F., Akoglu M. (2004). Comparison of complications after D2 and D3 dissection for gastric cancer. Eur. J. Surg. Oncol..

[47] Kunisaki C., Akiyama H., Nomura M., Matsuda G., Otsuka Y., Ono H., Nagahori Y., Hosoi H., Takahashi M., Kito F. (2006). Comparison of surgical results of D2 versus D3 gastrectomy (para-aortic lymph node dissection) for advanced gastric carcinoma: A multi-institutional study. Ann. Surg. Oncol..

[48] Marrelli D., Pedrazzani C., Neri A., Corso G., De Stefano A., Pinto E., Roviello F. (2007). Complications after extended (D2) and superextended (D3) lymphadenectomy for gastric cancer: Analysis of potential risk factors. Ann. Surg. Oncol..

[49] Eom B.W., Joo J., Kim Y.W., Park B., Park J.Y., Yoon H.M., Lee J.H., Ryu K.W. (2013). Is there any role of additional retropancreatic lymph node dissection on D2 gastrectomy for advanced gastric cancer?. Ann. Surg. Oncol..

[50] Eom B.W., Joo J., Kim Y.W., Reim D., Park J.Y., Yoon H.M., Ryu K.W., Lee J.Y., Kook M.C. (2014). Improved survival after adding dissection of the superior mesenteric vein lymph node (14v) to standard D2 gastrectomy for advanced distal gastric cancer. Surgery.

[51] Zhang Y., Tian S. (2013). Does D2 plus para-aortic nodal dissection surgery offer A better survival outcome compared to D2 surgery only for gastric cancer consistently? a definite result based on a hospital population of nearly two decades. Scand. J. Surg..

[52] Harrison L.E., Karpeh M.S., Brennan M.F. (1998). Extended Lymphadenectomy Is Associated with a Survival Benefit for Node-Negative Gastric Cancer. J. Gastrointest. Surg..

[53] Sierra A., Regueira F.M., Hern’Ndez-Lizo’In J.L., Pardo F., Martínez-Gonzalez M.A., Cienfuegos J.A. (2003). Role of the extended lymphadenectomy in gastric cancer surgery: Experience in a single institution. Ann. Surg. Oncol..

[54] Kasakura Y., Mochizuki F., Wakabayashi K., Kochi M., Fujii M., Takayama T. (2002). An evaluation of the effectiveness of extended lymph node dissection in patients with gastric cancer: A retrospective study of 1403 cases at a single institution. J. Surg. Res..

[55] Siewert J.R., Böttcher K., Roder J.D., Busch R., Hermanek P., Meyer H.J. (1993). Prognostic relevance of systematic lymph node dissection in gastric carcinoma. Br. J. Surg..

[56] Roukos D., Schmidt-Mathiesen A., Encke A. (1995). Adenocarcinoma of the gastric antrum: Does D2 total gastrectomy with splenectomy improve prognosis compared to D1 subtotal gastrectomy? A long-term survival analysis with emphasis on Lauren classification. Surg. Oncol..

[57] Galizia G., Lieto E., De Vita F., Castellano P., Ferraraccio F., Zamboli A., Mabilia A., Auricchio A., De Sena G., De Stefano L. (2015). Modified versus standard D2 lymphadenectomy in total gastrectomy for nonjunctional gastric carcinoma with lymph node metastasis. Surgery.

[58] Brenkman H.J.F., Goense L., Brosens L.A., Haj Mohammad N., Vleggaar F.P., Ruurda J.P., van Hillegersberg R. (2017). A High Lymph Node Yield is Associated with Prolonged Survival in Elderly Patients Undergoing Curative Gastrectomy for Cancer: A Dutch Population-Based Cohort Study. Ann. Surg. Oncol..

[59] Passot G., Vaudoyer D., Messager M., Brudvik K.W., Kim B.J., Mariette C., Glehen O. (2016). Is Extended Lymphadenectomy Needed for Elderly Patients with Gastric Adenocarcinoma?. Ann. Surg. Oncol..

[60] Pacelli F., Doglietto G.B., Bellantone R., Alfieri S., Sgadari A., Crucitti F. (1993). Extensive versus limited lymph ncde dissection for gastric cancer: A comparative study of 320 patients. Br. J. Surg..

[61] Edwards P., Blackshaw G.R.J.C., Lewis W.G., Barry J.D., Allison M.C., Jones D.R.B. (2004). Prospective comparison of D1 vs. modified D2 gastrectomy for carcinoma. Br. J. Cancer..

[62] Csendes A., Burdiles P., Rojas J., Braghetto I., Diaz J.C., Maluenda F. (2002). A prospective randomized study comparing D2 total gastrectomy versus D2 total gastrectomy plus splenectomy in 187 patients with gastric carcinoma. Surgery.

[63] Sasako M., Sano T., Yamamoto S., Kurokawa Y., Nashimoto A., Kurita A., Hiratsuka M., Tsujinaka T., Kinoshita T., Arai K. (2008). D2 Lymphadenectomy Alone or with Para-aortic Nodal Dissection for Gastric Cancer. N. Engl. J. Med..

[64] Sano T., Sasako M., Yamamoto S., Nashimoto A., Kurita A., Hiratsuka M., Tsujinaka T., Kinoshita T., Arai K., Yamamura Y. (2004). Gastric cancer surgery: Morbidity and mortality results from a prospective randomized controlled trial comparing D2 and extended para-aortic lymphadenectomy—Japan Clinical Oncology Group study 9501. J. Clin. Oncol..

[65] Maeta M., Yamashiro H., Saito H., Katano K., Kondo A., Tsujitani S., Ikeguchi M., Kaibara N. (1999). A prospective pilot study of extended (D3) and superextended para-aortic lymphadenectomy (D4) in patients with T3 or T4 gastric cancer managed by total gastrectomy. Surgery.

[66] Kulig J., Popiela T., Kolodziejczyk P., Sierzega M., Szczepanik A. (2007). Standard D2 versus extended D2 (D2^+^) lymphadenectomy for gastric cancer: An interim safety analysis of a multicenter, randomized, clinical trial. Am. J. Surg..

[67] Yonemura Y., Wu C.C., Fukushima N., Honda I., Bandou E., Kawamura T., Kamata T., Kim B.S., Matsuki N., Sawa T. (2008). Randomized clinical trial of D2 and extended paraaortic lymphadenectomy in patients with gastric cancer. Int. J. Clin. Oncol..

[68] Robertson C.S., Chung S.C.S., Woods S.D.S., Griffin S.M., Raimes S.A., Lau J.T.F., Li A.K.C. (1994). A prospective randomized trial comparing R1 subtotal gastrectomy with R3 total gastrectomy for antral cancer. Ann. Surg..

[69] Zhang H., Liu C., Wu D., Meng Y., Song R., Lu P., Wang S. (2010). Does D3 surgery offer a better survival outcome compared to D1 surgery for gastric cancer? A result based on a hospital population of two decades as taking D2 surgery for reference. BMC Cancer.

[70] Back J., Sallinen V., Kokkola A., Puolakkainen P. (2022). Surgical and oncological outcomes of D1 versus D2 gastrectomy among elderly patients treated for gastric cancer. Scand. J. Surg..

[71] Seo H.S., Jung Y.J., Kim J.H., Park C.H., Lee H.H. (2018). Necessity of D2 lymph node dissection in older patients ≥ 80 years with gastric cancer. J. Geriatr. Oncol..

[72] Mikami K., Hirano K., Futami K., Maekawa T. (2018). Gastrectomy with limited surgery for elderly patients with gastric cancer. Asian J. Surg..

[73] Cuschieri A., Fayers P., Fielding J., Craven J., Bancewicz J., Joypaul V., Cook P. (1996). Postoperative morbidity and mortality after D1 and D2 resections for gastric cancer: Preliminary results of the MRC randomised controlled surgical trial. Lancet.

[74] Cuschieri A., Weeden S., Fielding J., Bancewicz J., Craven J., Joypaul V., Sydes M., Fayers P. (1999). Patient survival after D1 and D2 resections for gastric cancer: Long-term results of the MRC randomized surgical trial. Br. J. Cancer..

[75] Bonenkamp J.J., Songun I., Welvaart K., van de Velde C.J.H., Hermans J., Sasako M., Plukker J.T.M., van Elk P., Obertop H., Gouma D.J. (1995). Randomised comparison of morbidity after D1 and D2 dissection for gastric cancer in 996 Dutch patients. Lancet.

[76] Bonenkamp J., Hermans J., Sasako M., van de Velde C.J.H. (1999). Extended lymph-node dissection for gastric cancer. N. Engl. J. Med..

[77] Degiuli M., Sasako M., Ponti A. (2010). Morbidity and mortality in the Italian gastric cancer study group randomized clinical trial of D1 versus D2 resection for gastric cancer. Br. J. Surg..

[78] Degiuli M., Sasako M., Ponti A., Vendrame A., Tomatis M., Mazza C., Borasi A., Capussotti L., Fronda G., Morino M. (2014). Randomized clinical trial comparing survival after D1 or D2 gastrectomy for gastric cancer. Br. J. Surg..

[79] Wu C.W., Hsiung C.A., Lo S.S., Hsieh M.C., Chen J.H., Li A.F.Y., Lui W.Y., Whang-Peng J. (2006). Nodal dissection for patients with gastric cancer: A randomised controlled trial. Lancet Oncol..

[80] Wu C.W., Hsiung C.A., Lo S.S., Hsieh M.C., Shia L.T., Whang-Peng J. (2004). Randomized clinical trial of morbidity after D1 and D3 surgery for gastric cancer. Br. J. Surg..

[81] Oñate-Ocaña L.F., Aiello-Crocifoglio V., Mondragón-Sánchez R., Ruiz-Molina J.M. (2000). Survival benefit of D2 lymphadenectomy in patients with gastric adenocarcinoma. Ann. Surg. Oncol..

[82] Lam S., Tan E., Menezes A., Martin D., Gallagher J., Storey D., Sandroussi C. (2018). A comparison of the operative outcomes of D1 and D2 gastrectomy performed at a single Western center with multiple surgeons: A retrospective analysis with propensity score matching. World J. Surg. Oncol..

[83] Wohnrath D.R., Araujo R.L.C. (2019). D2 lymphadenectomy for gastric cancer as an independent prognostic factor of 10-year overall survival. Eur. J. Surg. Oncol..

[84] Kang J.H., Ryu S.Y., Jung M.R., Jeong O. (2020). Comparison of long term survival outcomes between D1^+^ and D2 lymph node dissection for ≥pT2 or pN^+^ gastric carcinoma: A large scale case-control study using propensity score matching. Eur. J. Surg. Oncol..

[85] Ramos M.F.K.P., Pereira M.A., Dias A.R., Yagi O.K., Zaidan E.P., Ribeiro-Júnior U., Zilberstein B., Cecconello I. (2019). Surgical outcomes of gastrectomy with D1 lymph node dissection performed for patients with unfavorable clinical conditions. Eur. J. Surg. Oncol..

[86] Kota I., Makoto H., Satoshi K., Yutaka T., Etsuro B., Masanori T. (2021). Oncologic feasibility of D1+ gastrectomy for patients with cT1N1, cT2N0-1, or cT3N0 gastric cancer. Eur. J. Surg. Oncol..

[87] Rausei S., Ruspi L., Rosa F., Morgagni P., Marrelli D., Cossu A., Cananzi F.C.M., Lomonaco R., Coniglio A., Biondi A. (2016). Extended lymphadenectomy in elderly and/or highly co-morbid gastric cancer patients: A retrospective multicenter study. Eur. J. Surg. Oncol..

[88] Takama T., Okano K., Kondo A., Akamoto S., Fujiwara M., Usuki H., Suzuki Y. (2015). Predictors of postoperative complications in elderly and oldest old patients with gastric cancer. Gastric Cancer.

[89] Onagi C., Oba M., Oshima Y., Shimada H. (2022). Systematic review and meta-analysis of reports of patients with gastric cancer aged 80 years and older. Int. Cancer Conf. J..

[90] Mohanty S., Rosenthal R.A., Russell M.M., Neuman M.D., Ko C.Y., Esnaola N.F. (2016). Optimal Perioperative Management of the Geriatric Patient: A Best Practices Guideline from the American College of Surgeons NSQIP and the American Geriatrics Society. J. Am. Coll. Surg..

[91] Thillainadesan J., Hilmer S.N., Fleury A.M., Naganathan V. (2022). New horizons in the perioperative care of older adults. Age Ageing.

[92] Muhandiramge J., Orchard S.G., Warner E.T., van Londen G.J., Zalcberg J.R. (2022). Functional Decline in the Cancer Patient: A Review. Cancers.

[93] Amemiya T., Oda K., Ando M., Kawamura T., Kitagawa Y., Okawa Y., Yasui A., Ike H., Shimada H., Kuroiwa K. (2007). Activities of daily living and quality of life of elderly patients after elective surgery for gastric and colorectal cancers. Ann. Surg..

[94] Nunobe S., Oda I., Ishikawa T., Akazawa K., Katai H., Isobe Y., Miyashiro I., Tsujitani S., Ono H., Tanabe S. (2020). Surgical outcomes of elderly patients with Stage I gastric cancer from the nationwide registry of the Japanese Gastric Cancer Association. Gastric Cancer.

[95] Joharatnam-Hogan N., Shiu K.K., Khan K. (2020). Challenges in the treatment of gastric cancer in the older patient. Cancer Treat. Rev..

[96] Paredero-Pérez I., Jimenez-Fonseca P., Cano J.M., Arrazubi V., Carmona-Bayonas A., Covela-Rúa M., Fernández-Montes A., Martín-Richard M., Gironés-Sarrió R. (2023). State of the scientific evidence and recommendations for the management of older patients with gastric cancer. J. Geriatr. Oncol..

[97] Tan E., Lam S., Han S.P., Storey D., Sandroussi C. (2021). Perioperative outcomes and survival in elderly patients aged ≥75 years undergoing gastrectomy for gastric cancer: An 18-year retrospective analysis in a single Western centre. Langenbeck’s Arch. Surg..

[98] Guo J., Huang X., Dou L., Yan M., Shen T., Tang W., Li J. (2022). Aging and aging-related diseases: From molecular mechanisms to interventions and treatments. Signal Transduct. Target. Ther..

[99] Ruiz M., Reske T., Cefalu C., Estrada J. (2013). Management of elderly and frail elderly cancer patients: The importance of comprehensive geriatrics assessment and the need for guidelines. Am. J. Med. Sci..

[100] Marano L., Carbone L., Poto G.E., Gambelli M., Nguefack Noudem L.L., Grassi G., Manasci F., Curreri G., Giuliani A., Piagnerelli R. (2022). Handgrip strength predicts length of hospital stay in an abdominal surgical setting: The role of frailty beyond age. Aging Clin. Exp. Res..

