# Supplementary material for: Lymph Node Dissection of Choice in Older Adult Patients with Gastric Cancer: A Systematic Review and Meta-Analysis
Source: J Clin Med. 2024 Dec 17;13(24):7678. doi: 10.3390/jcm13247678 (PMC11678213; doi:10.3390/jcm13247678)
Supplement: Supplementary file 1 [file jcm-13-07678-s001.zip › S1. Quality assessment.pdf]

Supplement S1. Quality assessment of included studies.

a. Non-randomized comparative studies in elderly

Study	Risk Of Bias In Non-randomized Studies of Interventions (ROBINS-I) Domains							
	Confounding	Selection of participants	Classification of interventions	Deviations from interventions	Missing data	Measurement of outcomes	Reporting	Overall bias
Sakaguchi, et al.	Moderate	Serious	Lower	Moderate	Lower	Lower	Lower	Serious
Ko, et al.	Moderate	Moderate	Lower	No information	Lower	Lower	Lower	Moderate
Back, et al.	Serious	Moderate	Lower	No information	Lower	Lower	Lower	Serious
Shinozuka, et al.	Moderate	Moderate	Lower	No information	Lower	Lower	Lower	Moderate
Seo, et al.	Serious	Lower	Lower	No information	Lower	Lower	Lower	Serious
Mikami, et al.	Serious	Lower	Moderate	No information	Lower	Moderate	Lower	Serious

b. Randomized controlled trials

Study	Domain risk of bias (Cochrane Risk of Bias 2 tool for randomized trials)					
	Randomization process	Deviation from intervention	Missing outcome data	Outcome measurement	Selection of the reported result	Overall bias
MRC ST01	Lower	Some concerns	Lower	Lower	Some concerns	Lower
Dutch gastric cancer trial	Lower	Higher	Lower	Some concerns	Some concerns	Some concerns
Italian Gastric Cancer Study Group	Lower	Some concerns	Lower	Lower	Lower	Low
Wu	Lower	Higher	Lower	Some concerns	Some concerns	Some concerns

c. Non-randomized comparative studies

Study	Risk Of Bias In Non-randomized Studies of Interventions (ROBINS-I) Domains

	Confounding	Selection of participants	Classification of interventions	Deviations from interventions	Missing data	Measurement of outcomes	Reporting	Overall bias
Oñate-Ocaña, et al.	Serious	Critical	Lower	Lower	Lower	Lower	Lower	Serious
Zhang, et al.	Serious	Serious	Lower	No information	Lower	Lower	Lower	Serious
Susanna Lam, et al.	Moderate	Moderate	Serious	Lower	Lower	Lower	Lower	Serious
Wohnrath, et al.	Moderate	Serious	Lower	No information	Lower	Lower	Lower	Serious
Ji Hoon Kang, et al.	Serious	Serious	Lower	No information	Lower	Lower	Lower	Serious
Pertille Ramos, et al.	Serious	Serious	Serious	No information	Lower	Lower	Lower	Serious
Kota, et al.	Moderate	Serious	Moderate	Lower	Lower	Lower	Lower	Serious
