# Supplementary material for: Lymph Node Dissection of Choice in Older Adult Patients with Gastric Cancer: A Systematic Review and Meta-Analysis
Source: J Clin Med. 2024 Dec 17;13(24):7678. doi: 10.3390/jcm13247678 (PMC11678213; doi:10.3390/jcm13247678)
Supplement: Supplementary file 1 [file jcm-13-07678-s001.zip › S2. Sensitivity analysis.pdf]

S2. Sensitivity analysis

a. **Overall survival**

```
                        HR             95%-CI %W(random) exclude
Masazumi Sakaguchi 1.3366 [0.5600; 3.1900]        0.0       *
Chang Seok Ko      0.6478 [0.3861; 1.0870]       23.6
Johan Back         1.2355 [0.7100; 2.1500]       22.5
Takahiro Shinozuka 1.4952 [0.9200; 2.4300]       24.7
Ho Seok Seo        2.0030 [0.5900; 6.8000]        9.0
Koji Mikami        0.6205 [0.3311; 1.1628]       20.2

Number of studies: k = 5

                          HR             95%-CI    z p-value
Random effects model 1.0102 [0.6623; 1.5408] 0.05  0.9624

Quantifying heterogeneity:
 tau^2 = 0.1266 [0.0000; 1.9025]; tau = 0.3558 [0.0000; 1.3793]
 I^2 = 57.3% [0.0%; 84.1%]; H = 1.53 [1.00; 2.51]

Test of heterogeneity:
    Q d.f.  p-value
 9.36    4   0.0528
```

```
                        HR             95%-CI %W(random) exclude
Masazumi Sakaguchi 1.3366 [0.5600; 3.1900]       13.8
Chang Seok Ko      0.6478 [0.3861; 1.0870]        0.0       *
Johan Back         1.2355 [0.7100; 2.1500]       26.0
Takahiro Shinozuka 1.4952 [0.9200; 2.4300]       30.3
Ho Seok Seo        2.0030 [0.5900; 6.8000]        7.8
Koji Mikami        0.6205 [0.3311; 1.1628]       22.2

Number of studies: k = 5

                          HR             95%-CI    z p-value
Random effects model 1.1792 [0.8209; 1.6939] 0.89  0.3723

Quantifying heterogeneity:
 tau^2 = 0.0513 [0.0000; 1.3502]; tau = 0.2266 [0.0000; 1.1620]
 I^2 = 30.6% [0.0%; 73.3%]; H = 1.20 [1.00; 1.93]

Test of heterogeneity:
    Q d.f.  p-value
 5.76    4   0.2177
```

```
                        HR            95%-CI %W(random) exclude
Masazumi Sakaguchi 1.3366 [0.5600; 3.1900]       16.0        
Chang Seok Ko      0.6478 [0.3861; 1.0870]       25.4        
Johan Back         1.2355 [0.7100; 2.1500]        0.0       *
Takahiro Shinozuka 1.4952 [0.9200; 2.4300]       26.4        
Ho Seok Seo        2.0030 [0.5900; 6.8000]       10.2        
Koji Mikami        0.6205 [0.3311; 1.1628]       22.0        

Number of studies: k = 5

                         HR            95%-CI    z p-value
Random effects model 1.0084 [0.6373; 1.5956] 0.04  0.9714

Quantifying heterogeneity:
 tau^2 = 0.1463 [0.0000; 2.1012]; tau = 0.3825 [0.0000; 1.4496]
 I^2 = 56.5% [0.0%; 83.9%]; H = 1.52 [1.00; 2.49]

Test of heterogeneity:
    Q d.f. p-value
 9.20    4  0.0563

                        HR            95%-CI %W(random) exclude
Masazumi Sakaguchi 1.3366 [0.5600; 3.1900]       14.9        
Chang Seok Ko      0.6478 [0.3861; 1.0870]       27.7        
Johan Back         1.2355 [0.7100; 2.1500]       25.9        
Takahiro Shinozuka 1.4952 [0.9200; 2.4300]        0.0       *
Ho Seok Seo        2.0030 [0.5900; 6.8000]        8.8        
Koji Mikami        0.6205 [0.3311; 1.1628]       22.7        

Number of studies: k = 5

                         HR            95%-CI     z p-value
Random effects model 0.9329 [0.6273; 1.3871] -0.34  0.7313

Quantifying heterogeneity:
 tau^2 = 0.0781 [0.0000; 1.7599]; tau = 0.2794 [0.0000; 1.3266]
 I^2 = 39.3% [0.0%; 77.5%]; H = 1.28 [1.00; 2.11]

Test of heterogeneity:
    Q d.f. p-value
 6.59    4  0.1591
```

```
                        HR            95%-CI %W(random) exclude
Masazumi Sakaguchi 1.3366 [0.5600; 3.1900]       13.0
Chang Seok Ko      0.6478 [0.3861; 1.0870]       22.7
Johan Back         1.2355 [0.7100; 2.1500]       21.4
Takahiro Shinozuka 1.4952 [0.9200; 2.4300]       23.9
Ho Seok Seo        2.0030 [0.5900; 6.8000]        0.0       *
Koji Mikami        0.6205 [0.3311; 1.1628]       19.0

Number of studies: k = 5

                          HR            95%-CI     z p-value
Random effects model 0.9897 [0.6730; 1.4555] -0.05  0.9582

Quantifying heterogeneity:
 tau^2 = 0.1008 [0.0000; 1.4301]; tau = 0.3176 [0.0000; 1.1958]
 I^2 = 53.2% [0.0%; 82.8%]; H = 1.46 [1.00; 2.41]

Test of heterogeneity:
    Q d.f. p-value
 8.54    4  0.0736
```

```
                        HR            95%-CI %W(random) exclude
Masazumi Sakaguchi 1.3366 [0.5600; 3.1900]       13.8
Chang Seok Ko      0.6478 [0.3861; 1.0870]       26.1
Johan Back         1.2355 [0.7100; 2.1500]       24.3
Takahiro Shinozuka 1.4952 [0.9200; 2.4300]       27.7
Ho Seok Seo        2.0030 [0.5900; 6.8000]        8.1
Koji Mikami        0.6205 [0.3311; 1.1628]        0.0       *

Number of studies: k = 5

                          HR            95%-CI    z p-value
Random effects model 1.1572 [0.7917; 1.6913] 0.75  0.4509

Quantifying heterogeneity:
 tau^2 = 0.0741 [0.0000; 1.3569]; tau = 0.2723 [0.0000; 1.1648]
 I^2 = 41.2% [0.0%; 78.3%]; H = 1.30 [1.00; 2.15]

Test of heterogeneity:
    Q d.f. p-value
 6.80    4  0.1467
```

b. Relapse-free survival

```
                         HR           95%-CI %W(random) exclude
Masazumi Sakaguchi 0.9994 [0.4400; 2.2700]        0.0       *
Chang Seok Ko      0.4744 [0.2273; 0.9901]       29.1
Johan Back         1.1547 [0.6700; 1.9900]       38.5
Takahiro Shinozuka 1.1445 [0.5900; 2.2200]       32.4

Number of studies: k = 3

                           HR           95%-CI     z p-value
Random effects model 0.8891 [0.5191; 1.5227] -0.43  0.6684

Quantifying heterogeneity:
 tau^2 = 0.1184 [0.0000; 9.5477]; tau = 0.3441 [0.0000; 3.0899]
 I^2 = 52.4% [0.0%; 86.3%]; H = 1.45 [1.00; 2.70]

Test of heterogeneity:
    Q d.f. p-value
 4.20    2  0.1225
```

```
                         HR           95%-CI %W(random) exclude
Masazumi Sakaguchi 0.9994 [0.4400; 2.2700]       20.8
Chang Seok Ko      0.4744 [0.2273; 0.9901]        0.0       *
Johan Back         1.1547 [0.6700; 1.9900]       47.3
Takahiro Shinozuka 1.1445 [0.5900; 2.2200]       31.9

Number of studies: k = 3

                           HR           95%-CI    z p-value
Random effects model 1.1173 [0.7685; 1.6245] 0.58  0.5613

Quantifying heterogeneity:
 tau^2 = 0 [0.0000; 0.1139]; tau = 0 [0.0000; 0.3374]
 I^2 = 0.0% [0.0%; 89.6%]; H = 1.00 [1.00; 3.10]

Test of heterogeneity:
    Q d.f. p-value
 0.09    2  0.9560
```

```
                           HR            95%-CI %W(random) exclude
Masazumi Sakaguchi 0.9994 [0.4400; 2.2700]       29.1
Chang Seok Ko      0.4744 [0.2273; 0.9901]       33.3
Johan Back         1.1547 [0.6700; 1.9900]        0.0        *
Takahiro Shinozuka 1.1445 [0.5900; 2.2200]       37.6

Number of studies: k = 3

                             HR            95%-CI     z p-value
Random effects model 0.8204 [0.4745; 1.4185] -0.71  0.4786

Quantifying heterogeneity:
 tau^2 = 0.0933 [0.0000; 8.9326]; tau = 0.3054 [0.0000; 2.9887]
 I^2 = 39.8% [0.0%; 81.4%]; H = 1.29 [1.00; 2.32]

Test of heterogeneity:
    Q d.f. p-value
 3.32    2  0.1901
```

```
                           HR            95%-CI %W(random) exclude
Masazumi Sakaguchi 0.9994 [0.4400; 2.2700]       27.3
Chang Seok Ko      0.4744 [0.2273; 0.9901]       31.0
Johan Back         1.1547 [0.6700; 1.9900]       41.6
Takahiro Shinozuka 1.1445 [0.5900; 2.2200]        0.0        *

Number of studies: k = 3

                             HR            95%-CI     z p-value
Random effects model 0.8422 [0.4875; 1.4549] -0.62  0.5380

Quantifying heterogeneity:
 tau^2 = 0.1097 [0.0000; 9.0209]; tau = 0.3312 [0.0000; 3.0035]
 I^2 = 46.8% [0.0%; 84.4%]; H = 1.37 [1.00; 2.53]

Test of heterogeneity:
    Q d.f. p-value
 3.76    2  0.1528
```

c. **Cancer-specific survival**

```
                          HR            95%-CI %W(random) exclude
Masazumi Sakaguchi 2.6965 [0.4700; 15.4700]        0.0       *
Johan Back         1.2185 [0.5800;  2.5600]       33.6
Takahiro Shinozuka 1.7440 [0.7900;  3.8500]       31.1
Ho Seok Seo        1.7693 [0.4300;  7.2800]       13.4
Koji Mikami        0.4618 [0.1642;  1.2987]       21.9

Number of studies: k = 4

                            HR           95%-CI    z p-value
Random effects model 1.1586 [0.6552; 2.0486] 0.51  0.6127

Quantifying heterogeneity:
 tau^2 = 0.1084 [0.0000; 4.9490]; tau = 0.3293 [0.0000; 2.2246]
 I^2 = 32.1% [0.0%; 75.8%]; H = 1.21 [1.00; 2.03]

Test of heterogeneity:
    Q d.f.  p-value
 4.42    3  0.2196


                          HR            95%-CI %W(random) exclude
Masazumi Sakaguchi 2.6965 [0.4700; 15.4700]       14.7
Johan Back         1.2185 [0.5800;  2.5600]        0.0       *
Takahiro Shinozuka 1.7440 [0.7900;  3.8500]       36.7
Ho Seok Seo        1.7693 [0.4300;  7.2800]       19.8
Koji Mikami        0.4618 [0.1642;  1.2987]       28.8

Number of studies: k = 4

                            HR           95%-CI    z p-value
Random effects model 1.2716 [0.5883; 2.7482] 0.61  0.5412

Quantifying heterogeneity:
 tau^2 = 0.2585 [0.0000; 8.0652]; tau = 0.5084 [0.0000; 2.8399]
 I^2 = 42.5% [0.0%; 80.7%]; H = 1.32 [1.00; 2.28]

Test of heterogeneity:
    Q d.f.  p-value
 5.22    3  0.1566
```

```
                       HR            95%-CI %W(random) exclude
Masazumi Sakaguchi 2.6965 [0.4700; 15.4700]       12.5        
Johan Back         1.2185 [0.5800;  2.5600]       41.6        
Takahiro Shinozuka 1.7440 [0.7900;  3.8500]        0.0       *
Ho Seok Seo        1.7693 [0.4300;  7.2800]       17.7        
Koji Mikami        0.4618 [0.1642;  1.2987]       28.1        

Number of studies: k = 4

                         HR            95%-CI    z p-value
Random effects model 1.0949 [0.5605; 2.1385] 0.27  0.7908

Quantifying heterogeneity:
 tau^2 = 0.1368 [0.0000; 7.0680]; tau = 0.3699 [0.0000; 2.6586]
 I^2 = 28.8% [0.0%; 73.8%]; H = 1.19 [1.00; 1.95]

Test of heterogeneity:
    Q d.f.  p-value
 4.22    3  0.2391
```

```
                       HR            95%-CI %W(random) exclude
Masazumi Sakaguchi 2.6965 [0.4700; 15.4700]       10.8        
Johan Back         1.2185 [0.5800;  2.5600]       33.9        
Takahiro Shinozuka 1.7440 [0.7900;  3.8500]       31.8        
Ho Seok Seo        1.7693 [0.4300;  7.2800]        0.0       *
Koji Mikami        0.4618 [0.1642;  1.2987]       23.5        

Number of studies: k = 4

                         HR            95%-CI    z p-value
Random effects model 1.1849 [0.6325; 2.2195] 0.53  0.5963

Quantifying heterogeneity:
 tau^2 = 0.1588 [0.0000; 6.5098]; tau = 0.3984 [0.0000; 2.5514]
 I^2 = 39.5% [0.0%; 79.4%]; H = 1.29 [1.00; 2.20]

Test of heterogeneity:
    Q d.f.  p-value
 4.96    3  0.1748
```

```
                         HR             95%-CI %W(random) exclude
Masazumi Sakaguchi 2.6965 [0.4700; 15.4700]        7.7
Johan Back         1.2185 [0.5800;  2.5600]       42.8
Takahiro Shinozuka 1.7440 [0.7900;  3.8500]       37.6
Ho Seok Seo        1.7693 [0.4300;  7.2800]       11.8
Koji Mikami        0.4618 [0.1642;  1.2987]        0.0       *

Number of studies: k = 4

                           HR           95%-CI    z p-value
Random effects model 1.5496 [0.9533; 2.5190] 1.77  0.0772

Quantifying heterogeneity:
 tau^2 = 0 [0.0000; 1.0104]; tau = 0 [0.0000; 1.0052]
 I^2 = 0.0% [0.0%; 84.7%]; H = 1.00 [1.00; 2.56]

Test of heterogeneity:
    Q d.f.  p-value
 0.91    3   0.8234
```

d. Postoperative complications (Clavien-Dindo ≥ 3)

```
                        OR             95%-CI %W(common) %W(random) exclude
Masazumi Sakaguchi 10.3186 [0.5249; 202.8272]        0.0        0.0       *
Chang Seok Ko       2.3629 [0.5946;   9.3900]        6.9       16.8
Johan Back          1.4636 [0.6500;   3.2955]       22.2       21.6
Takahiro Shinozuka  1.5041 [0.5783;   3.9122]       16.0       20.4
Ho Seok Seo         8.5262 [2.7838;  26.1132]        5.1       19.0
Koji Mikami         0.4169 [0.2011;   0.8642]       49.7       22.2

Number of studies: k = 5
Number of observations: o = 932
Number of events: e = 128.8

                         OR           95%-CI    z p-value
Common effect model  1.3741 [0.9293; 2.0320] 1.59  0.1113
Random effects model 1.6864 [0.6384; 4.4543] 1.05  0.2917

Quantifying heterogeneity:
 tau^2 = 0.9664 [0.1901; 9.4767]; tau = 0.9831 [0.4360; 3.0784]
 I^2 = 81.0% [55.8%; 91.9%]; H = 2.30 [1.50; 3.51]

Test of heterogeneity:
     Q d.f.  p-value
 21.09    4   0.0003

Details on meta-analytical method:
- Mantel-Haenszel method
- Inverse variance method
- Restricted maximum-likelihood estimator for tau^2
```

```
                         OR             95%-CI %W(common) %W(random) exclude
Masazumi Sakaguchi 10.3186 [0.5249; 202.8272]        1.0        9.4        
Chang Seok Ko       2.3629 [0.5946;   9.3900]        0.0        0.0       *
Johan Back          1.4636 [0.6500;   3.2955]       23.6       23.3        
Takahiro Shinozuka  1.5041 [0.5783;   3.9122]       17.0       22.3        
Ho Seok Seo         8.5262 [2.7838;  26.1132]        5.5       21.1        
Koji Mikami         0.4169 [0.2011;   0.8642]       52.9       23.9        

Number of studies: k = 5
Number of observations: o = 932
Number of events: e = 128.8

                         OR           95%-CI    z p-value
Common effect model  1.3939 [0.9329; 2.0827] 1.62  0.1050
Random effects model 1.8990 [0.6109; 5.9034] 1.11  0.2678

Quantifying heterogeneity:
 tau^2 = 1.2631 [0.2385; 14.2116]; tau = 1.1239 [0.4884; 3.7698]
 I^2 = 82.0% [58.5%; 92.2%]; H = 2.36 [1.55; 3.58]

Test of heterogeneity:
     Q d.f. p-value
 22.22    4  0.0002
```

```
                         OR             95%-CI %W(common) %W(random) exclude
Masazumi Sakaguchi 10.3186 [0.5249; 202.8272]        1.2       10.0        
Chang Seok Ko       2.3629 [0.5946;   9.3900]        8.7       20.0        
Johan Back          1.4636 [0.6500;   3.2955]        0.0        0.0       *
Takahiro Shinozuka  1.5041 [0.5783;   3.9122]       20.4       23.2        
Ho Seok Seo         8.5262 [2.7838;  26.1132]        6.5       22.0        
Koji Mikami         0.4169 [0.2011;   0.8642]       63.2       24.8        

Number of studies: k = 5
Number of observations: o = 932
Number of events: e = 128.8

                         OR           95%-CI    z p-value
Common effect model  1.4586 [0.9428; 2.2565] 1.70  0.0900
Random effects model 2.1284 [0.6494; 6.9756] 1.25  0.2123

Quantifying heterogeneity:
 tau^2 = 1.3418 [0.2456; 13.6682]; tau = 1.1584 [0.4956; 3.6971]
 I^2 = 82.5% [59.9%; 92.4%]; H = 2.39 [1.58; 3.62]

Test of heterogeneity:
     Q d.f. p-value
 22.86    4  0.0001
```

```
                             OR             95%-CI %W(common) %W(random) exclude
Masazumi Sakaguchi 10.3186 [0.5249; 202.8272]        1.1        9.9
Chang Seok Ko       2.3629 [0.5946;   9.3900]        8.1       19.8
Johan Back          1.4636 [0.6500;   3.2955]       26.2       24.0
Takahiro Shinozuka  1.5041 [0.5783;   3.9122]        0.0        0.0       *
Ho Seok Seo         8.5262 [2.7838;  26.1132]        6.1       21.8
Koji Mikami         0.4169 [0.2011;   0.8642]       58.6       24.6

Number of studies: k = 5
Number of observations: o = 932
Number of events: e = 128.8

                              OR           95%-CI    z p-value
Common effect model   1.4513 [0.9538; 2.2083] 1.74  0.0820
Random effects model 2.1054 [0.6484; 6.8367] 1.24  0.2154

Quantifying heterogeneity:
 tau^2 = 1.3326 [0.2466; 13.7338]; tau = 1.1544 [0.4966; 3.7059]
 I^2 = 82.5% [59.9%; 92.4%]; H = 2.39 [1.58; 3.62]

Test of heterogeneity:
     Q d.f. p-value
 22.85    4  0.0001


                             OR             95%-CI %W(common) %W(random) exclude
Masazumi Sakaguchi 10.3186 [0.5249; 202.8272]        1.0        5.8
Chang Seok Ko       2.3629 [0.5946;   9.3900]        7.2       17.1
Johan Back          1.4636 [0.6500;   3.2955]       23.2       26.1
Takahiro Shinozuka  1.5041 [0.5783;   3.9122]       16.7       23.5
Ho Seok Seo         8.5262 [2.7838;  26.1132]        0.0        0.0       *
Koji Mikami         0.4169 [0.2011;   0.8642]       51.9       27.5

Number of studies: k = 5
Number of observations: o = 932
Number of events: e = 128.8

                              OR           95%-CI    z p-value
Common effect model   1.0806 [0.7081; 1.6490] 0.36  0.7193
Random effects model 1.2670 [0.5784; 2.7755] 0.59  0.5541

Quantifying heterogeneity:
 tau^2 = 0.4427 [0.0000; 9.7293]; tau = 0.6654 [0.0000; 3.1192]
 I^2 = 63.4% [3.3%; 86.1%]; H = 1.65 [1.02; 2.69]

Test of heterogeneity:
     Q d.f. p-value
 10.92    4  0.0274
```

```
                                OR              95%-CI %W(common) %W(random) exclude
Masazumi Sakaguchi 10.3186 [0.5249; 202.8272]        1.9        6.0        
Chang Seok Ko       2.3629 [0.5946;   9.3900]       13.4       18.1        
Johan Back          1.4636 [0.6500;   3.2955]       43.4       28.3        
Takahiro Shinozuka  1.5041 [0.5783;   3.9122]       31.3       25.4        
Ho Seok Seo         8.5262 [2.7838;  26.1132]       10.0       22.3        
Koji Mikami         0.4169 [0.2011;   0.8642]        0.0        0.0       *

Number of studies: k = 5
Number of observations: o = 932
Number of events: e = 128.8

                         OR           95%-CI    z p-value
Common effect model  2.4724 [1.5324; 3.9890] 3.71  0.0002
Random effects model 2.6734 [1.2166; 5.8748] 2.45  0.0144

Quantifying heterogeneity:
 tau^2 = 0.3981 [0.0000; 6.5198]; tau = 0.6309 [0.0000; 2.5534]
 I^2 = 51.1% [0.0%; 82.1%]; H = 1.43 [1.00; 2.36]

Test of heterogeneity:
    Q d.f. p-value
 8.18    4  0.0850
```

e. Age as a risk factor for OS

```
                       HR           95%-CI %W(random) exclude
Cuschieri          1.0249 [1.0100; 1.0400]        0.0       *
Bonenkamp/Songun   0.8816 [0.6700; 1.1600]        0.5        
Degiuli            1.0247 [1.0000; 1.0500]       21.7        
Wu                 1.2554 [0.8000; 1.9700]        0.2        
Oñate-Ocaña        1.0198 [1.0000; 1.0400]       24.7        
Zhang              1.0105 [0.7400; 1.3800]        0.4        
Susanna Lam        0.6541 [0.2577; 1.6600]        0.0        
Wohnrath           1.4145 [1.0700; 1.8700]        0.5        
Kang               1.0350 [1.0300; 1.0400]       32.3        
Pertille Ramos     1.6073 [1.0900; 2.3700]        0.2        
Kota               1.0696 [1.0400; 1.1000]       19.5        

Number of studies: k = 10

                         HR           95%-CI    z p-value
Random effects model 1.0375 [1.0176; 1.0579] 3.72  0.0002

Quantifying heterogeneity:
 tau^2 = 0.0003 [0.0000; 0.0301]; tau = 0.0173 [0.0000; 0.1736]
 I^2 = 56.8% [12.5%; 78.7%]; H = 1.52 [1.07; 2.16]

Test of heterogeneity:
     Q d.f. p-value
 20.82    9  0.0135
```

```
                    HR            95%-CI %W(random) exclude
Cuschieri        1.0249 [1.0100; 1.0400]       22.5        
Bonenkamp/Songun 0.8816 [0.6700; 1.1600]        0.0       *
Degiuli          1.0247 [1.0000; 1.0500]       15.7        
Wu               1.2554 [0.8000; 1.9700]        0.1        
Oñate-Ocaña      1.0198 [1.0000; 1.0400]       18.8        
Zhang            1.0105 [0.7400; 1.3800]        0.2        
Susanna Lam      0.6541 [0.2577; 1.6600]        0.0        
Wohnrath         1.4145 [1.0700; 1.8700]        0.3        
Kang             1.0350 [1.0300; 1.0400]       28.6        
Pertille Ramos   1.6073 [1.0900; 2.3700]        0.1        
Kota             1.0696 [1.0400; 1.1000]       13.6        

Number of studies: k = 10

                         HR            95%-CI    z  p-value
Random effects model 1.0343 [1.0198; 1.0490] 4.68 < 0.0001

Quantifying heterogeneity:
 tau^2 = 0.0002 [0.0000; 0.0185]; tau = 0.0132 [0.0000; 0.1359]
 I^2 = 57.2% [13.5%; 78.8%]; H = 1.53 [1.08; 2.17]

Test of heterogeneity:
     Q d.f. p-value
 21.04    9  0.0125
```

```
                    HR            95%-CI %W(random) exclude
Cuschieri        1.0249 [1.0100; 1.0400]       26.4        
Bonenkamp/Songun 0.8816 [0.6700; 1.1600]        0.4        
Degiuli          1.0247 [1.0000; 1.0500]        0.0       *
Wu               1.2554 [0.8000; 1.9700]        0.1        
Oñate-Ocaña      1.0198 [1.0000; 1.0400]       22.8        
Zhang            1.0105 [0.7400; 1.3800]        0.3        
Susanna Lam      0.6541 [0.2577; 1.6600]        0.0        
Wohnrath         1.4145 [1.0700; 1.8700]        0.4        
Kang             1.0350 [1.0300; 1.0400]       32.3        
Pertille Ramos   1.6073 [1.0900; 2.3700]        0.2        
Kota             1.0696 [1.0400; 1.1000]       17.2        

Number of studies: k = 10

                         HR            95%-CI    z  p-value
Random effects model 1.0361 [1.0189; 1.0536] 4.15 < 0.0001

Quantifying heterogeneity:
 tau^2 = 0.0002 [0.0000; 0.0276]; tau = 0.0148 [0.0000; 0.1662]
 I^2 = 58.7% [16.9%; 79.5%]; H = 1.56 [1.10; 2.21]

Test of heterogeneity:
     Q d.f. p-value
 21.80    9  0.0095
```

```
                      HR            95%-CI %W(random) exclude
Cuschieri         1.0249 [1.0100; 1.0400]       22.4
Bonenkamp/Songun 0.8816 [0.6700; 1.1600]        0.3
Degiuli           1.0247 [1.0000; 1.0500]       15.8
Wu                1.2554 [0.8000; 1.9700]        0.0        *
Oñate-Ocaña       1.0198 [1.0000; 1.0400]       18.9
Zhang             1.0105 [0.7400; 1.3800]        0.2
Susanna Lam       0.6541 [0.2577; 1.6600]        0.0
Wohnrath          1.4145 [1.0700; 1.8700]        0.3
Kang              1.0350 [1.0300; 1.0400]       28.2
Pertille Ramos    1.6073 [1.0900; 2.3700]        0.1
Kota              1.0696 [1.0400; 1.1000]       13.8

Number of studies: k = 10

                         HR            95%-CI    z  p-value
Random effects model 1.0337 [1.0190; 1.0487] 4.54 < 0.0001

Quantifying heterogeneity:
 tau^2 = 0.0002 [0.0000; 0.0190]; tau = 0.0135 [0.0000; 0.1378]
 I^2 = 58.4% [16.2%; 79.3%]; H = 1.55 [1.09; 2.20]
```

```
                      HR            95%-CI %W(random) exclude
Cuschieri         1.0249 [1.0100; 1.0400]       27.4
Bonenkamp/Songun 0.8816 [0.6700; 1.1600]        0.4
Degiuli           1.0247 [1.0000; 1.0500]       20.1
Wu                1.2554 [0.8000; 1.9700]        0.1
Oñate-Ocaña       1.0198 [1.0000; 1.0400]        0.0        *
Zhang             1.0105 [0.7400; 1.3800]        0.3
Susanna Lam       0.6541 [0.2577; 1.6600]        0.0
Wohnrath          1.4145 [1.0700; 1.8700]        0.4
Kang              1.0350 [1.0300; 1.0400]       33.5
Pertille Ramos    1.6073 [1.0900; 2.3700]        0.2
Kota              1.0696 [1.0400; 1.1000]       17.7

Number of studies: k = 10

                         HR            95%-CI    z  p-value
Random effects model 1.0376 [1.0202; 1.0553] 4.28 < 0.0001

Quantifying heterogeneity:
 tau^2 = 0.0002 [0.0000; 0.0284]; tau = 0.0147 [0.0000; 0.1686]
 I^2 = 55.8% [10.2%; 78.2%]; H = 1.50 [1.06; 2.14]

Test of heterogeneity:
     Q d.f.  p-value
 20.35    9   0.0159
```

```
                        HR            95%-CI %W(random)  exclude
Cuschieri          1.0249 [1.0100; 1.0400]       22.3
Bonenkamp/Songun 0.8816 [0.6700; 1.1600]        0.3
Degiuli            1.0247 [1.0000; 1.0500]       16.0
Wu                 1.2554 [0.8000; 1.9700]        0.1
Oñate-Ocaña        1.0198 [1.0000; 1.0400]       19.0
Zhang              1.0105 [0.7400; 1.3800]        0.0        *
Susanna Lam        0.6541 [0.2577; 1.6600]        0.0
Wohnrath           1.4145 [1.0700; 1.8700]        0.3
Kang               1.0350 [1.0300; 1.0400]       27.9
Pertille Ramos     1.6073 [1.0900; 2.3700]        0.1
Kota               1.0696 [1.0400; 1.1000]       14.0

Number of studies: k = 10

                         HR             95%-CI    z  p-value
Random effects model 1.0341 [1.0191; 1.0493] 4.49 < 0.0001

Quantifying heterogeneity:
 tau^2 = 0.0002 [0.0000; 0.0205]; tau = 0.0139 [0.0000; 0.1432]
 I^2 = 59.7% [19.1%; 79.9%]; H = 1.57 [1.11; 2.23]

Test of heterogeneity:
     Q d.f. p-value
 22.32    9  0.0079
```

```
                        HR            95%-CI %W(random)  exclude
Cuschieri          1.0249 [1.0100; 1.0400]       22.4
Bonenkamp/Songun 0.8816 [0.6700; 1.1600]        0.3
Degiuli            1.0247 [1.0000; 1.0500]       15.8
Wu                 1.2554 [0.8000; 1.9700]        0.1
Oñate-Ocaña        1.0198 [1.0000; 1.0400]       18.8
Zhang              1.0105 [0.7400; 1.3800]        0.2
Susanna Lam        0.6541 [0.2577; 1.6600]        0.0        *
Wohnrath           1.4145 [1.0700; 1.8700]        0.3
Kang               1.0350 [1.0300; 1.0400]       28.3
Pertille Ramos     1.6073 [1.0900; 2.3700]        0.1
Kota               1.0696 [1.0400; 1.1000]       13.7

Number of studies: k = 10

                         HR             95%-CI    z  p-value
Random effects model 1.0340 [1.0194; 1.0489] 4.60 < 0.0001

Quantifying heterogeneity:
 tau^2 = 0.0002 [0.0000; 0.0171]; tau = 0.0134 [0.0000; 0.1306]
 I^2 = 58.0% [15.2%; 79.2%]; H = 1.54 [1.09; 2.19]

Test of heterogeneity:
     Q d.f. p-value
 21.41    9  0.0110
```

```
                   HR           95%-CI %W(random) exclude
Cuschieri        1.0249 [1.0100; 1.0400]       22.7
Bonenkamp/Songun 0.8816 [0.6700; 1.1600]        0.2
Degiuli          1.0247 [1.0000; 1.0500]       14.6
Wu               1.2554 [0.8000; 1.9700]        0.1
Oñate-Ocaña      1.0198 [1.0000; 1.0400]       18.2
Zhang            1.0105 [0.7400; 1.3800]        0.2
Susanna Lam      0.6541 [0.2577; 1.6600]        0.0
Wohnrath         1.4145 [1.0700; 1.8700]        0.0       *
Kang             1.0350 [1.0300; 1.0400]       31.4
Pertille Ramos   1.6073 [1.0900; 2.3700]        0.1
Kota             1.0696 [1.0400; 1.1000]       12.4

Number of studies: k = 10

                         HR           95%-CI    z  p-value
Random effects model 1.0328 [1.0199; 1.0458] 5.05 < 0.0001

Quantifying heterogeneity:
 tau^2 = 0.0001 [0.0000; 0.0132]; tau = 0.0111 [0.0000; 0.1150]
 I^2 = 48.5% [0.0%; 75.1%]; H = 1.39 [1.00; 2.01]

Test of heterogeneity:
     Q d.f. p-value
 17.49    9  0.0416

                   HR           95%-CI %W(random) exclude
Cuschieri        1.0249 [1.0100; 1.0400]       27.5
Bonenkamp/Songun 0.8816 [0.6700; 1.1600]        0.7
Degiuli          1.0247 [1.0000; 1.0500]       23.1
Wu               1.2554 [0.8000; 1.9700]        0.3
Oñate-Ocaña      1.0198 [1.0000; 1.0400]       25.4
Zhang            1.0105 [0.7400; 1.3800]        0.6
Susanna Lam      0.6541 [0.2577; 1.6600]        0.1
Wohnrath         1.4145 [1.0700; 1.8700]        0.7
Kang             1.0350 [1.0300; 1.0400]        0.0       *
Pertille Ramos   1.6073 [1.0900; 2.3700]        0.4
Kota             1.0696 [1.0400; 1.1000]       21.4

Number of studies: k = 10

                         HR           95%-CI    z p-value
Random effects model 1.0360 [1.0121; 1.0604] 2.97  0.0030

Quantifying heterogeneity:
 tau^2 = 0.0005 [0.0000; 0.0347]; tau = 0.0214 [0.0000; 0.1863]
 I^2 = 58.0% [15.4%; 79.2%]; H = 1.54 [1.09; 2.19]

Test of heterogeneity:
     Q d.f. p-value
 21.45    9  0.0108
```

```
                          HR             95%-CI %W(random) exclude
Cuschieri         1.0249 [1.0100; 1.0400]       22.7
Bonenkamp/Songun 0.8816 [0.6700; 1.1600]        0.2
Degiuli           1.0247 [1.0000; 1.0500]       14.6
Wu                1.2554 [0.8000; 1.9700]        0.1
Oñate-Ocaña       1.0198 [1.0000; 1.0400]       18.2
Zhang             1.0105 [0.7400; 1.3800]        0.2
Susanna Lam       0.6541 [0.2577; 1.6600]        0.0
Wohnrath          1.4145 [1.0700; 1.8700]        0.2
Kang              1.0350 [1.0300; 1.0400]       31.5
Pertille Ramos    1.6073 [1.0900; 2.3700]        0.0       *
Kota              1.0696 [1.0400; 1.1000]       12.3

Number of studies: k = 10

                              HR             95%-CI    z  p-value
Random effects model 1.0329 [1.0202; 1.0459] 5.10 < 0.0001

Quantifying heterogeneity:
 tau^2 = 0.0001 [0.0000; 0.0104]; tau = 0.0110 [0.0000; 0.1020]
 I^2 = 48.2% [0.0%; 75.0%]; H = 1.39 [1.00; 2.00]

Test of heterogeneity:
     Q d.f.  p-value
 17.38    9   0.0431
```

```
                          HR             95%-CI %W(random) exclude
Cuschieri         1.0249 [1.0100; 1.0400]       25.9
Bonenkamp/Songun 0.8816 [0.6700; 1.1600]        0.2
Degiuli           1.0247 [1.0000; 1.0500]       16.7
Wu                1.2554 [0.8000; 1.9700]        0.1
Oñate-Ocaña       1.0198 [1.0000; 1.0400]       20.8
Zhang             1.0105 [0.7400; 1.3800]        0.2
Susanna Lam       0.6541 [0.2577; 1.6600]        0.0
Wohnrath          1.4145 [1.0700; 1.8700]        0.2
Kang              1.0350 [1.0300; 1.0400]       35.7
Pertille Ramos    1.6073 [1.0900; 2.3700]        0.1
Kota              1.0696 [1.0400; 1.1000]        0.0       *

Number of studies: k = 10

                              HR             95%-CI    z  p-value
Random effects model 1.0284 [1.0147; 1.0423] 4.10 < 0.0001

Quantifying heterogeneity:
 tau^2 = 0.0001 [0.0000; 0.0260]; tau = 0.0112 [0.0000; 0.1612]
 I^2 = 45.7% [0.0%; 73.9%]; H = 1.36 [1.00; 1.96]

Test of heterogeneity:
     Q d.f.  p-value
 16.59    9   0.0556
```
