# Supplementary material for: Lymph Node Dissection of Choice in Older Adult Patients with Gastric Cancer: A Systematic Review and Meta-Analysis
Source: J Clin Med. 2024 Dec 17;13(24):7678. doi: 10.3390/jcm13247678 (PMC11678213; doi:10.3390/jcm13247678)
Supplement: Supplementary file 1 [file jcm-13-07678-s001.zip › S3. Funnel plot.pdf]

S3. Funnel plot for publication bias assessment

a. **Overall survival**

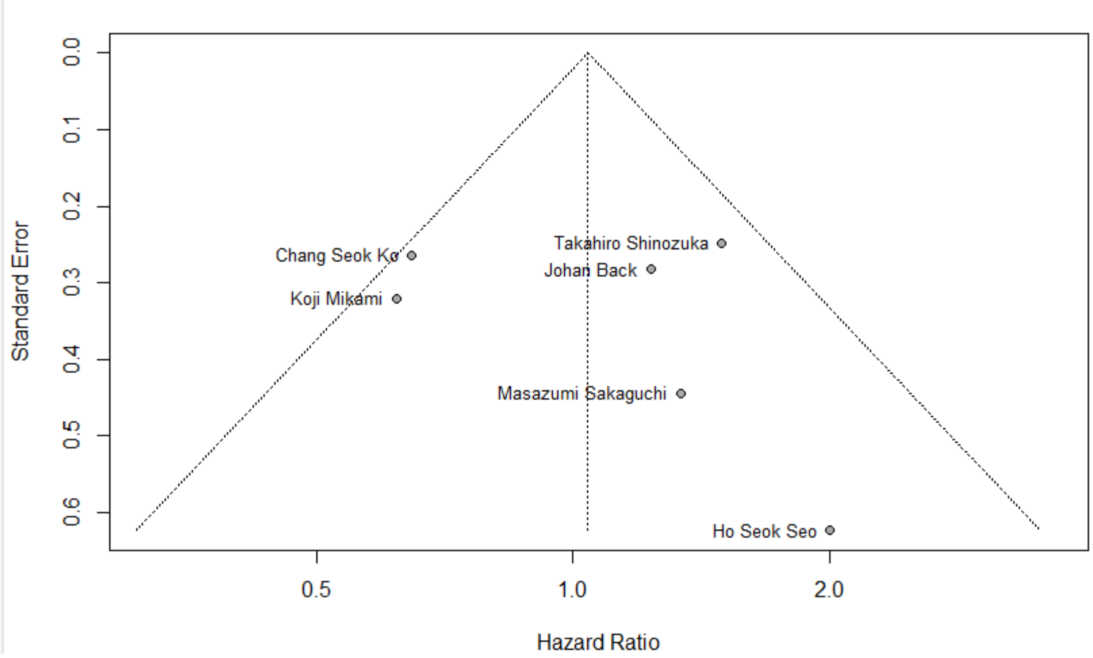

b. **Relapse-free survival**

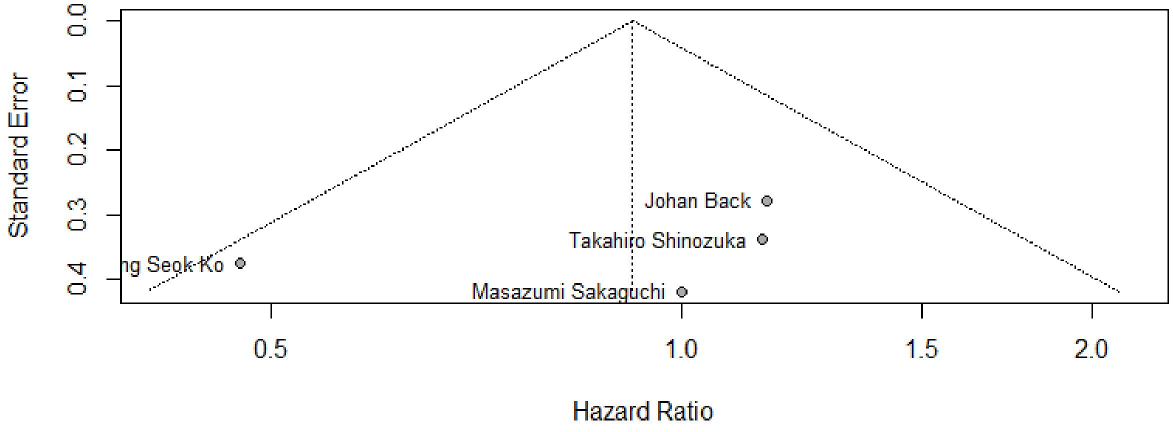

c. **Cancer-specific survival**

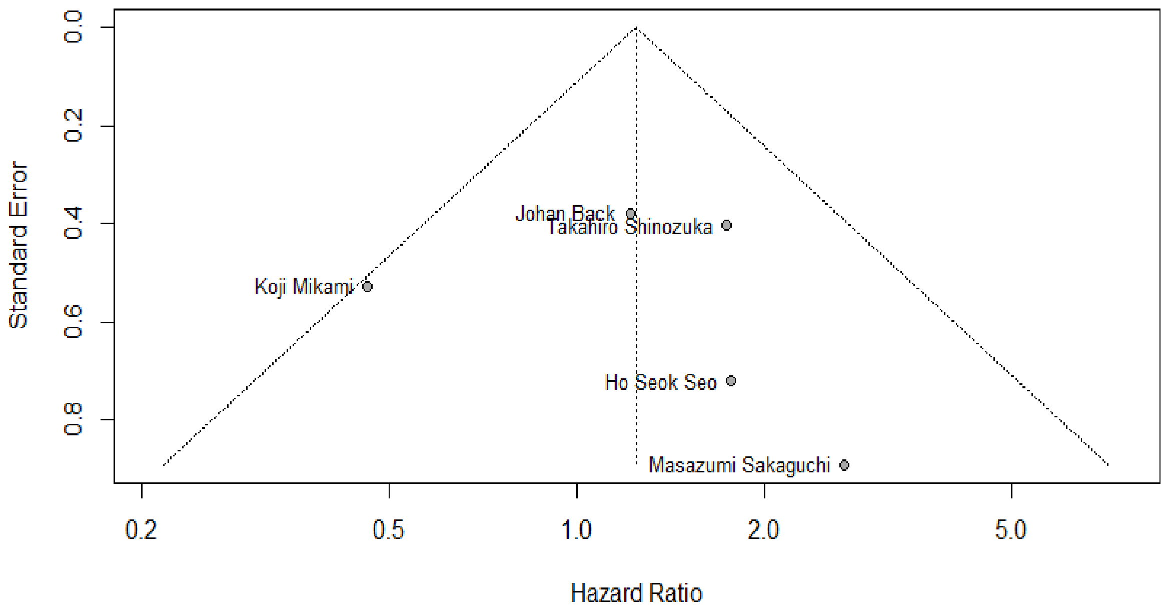

d. Postoperative complications (Clavien-Dindo ≥ 3)

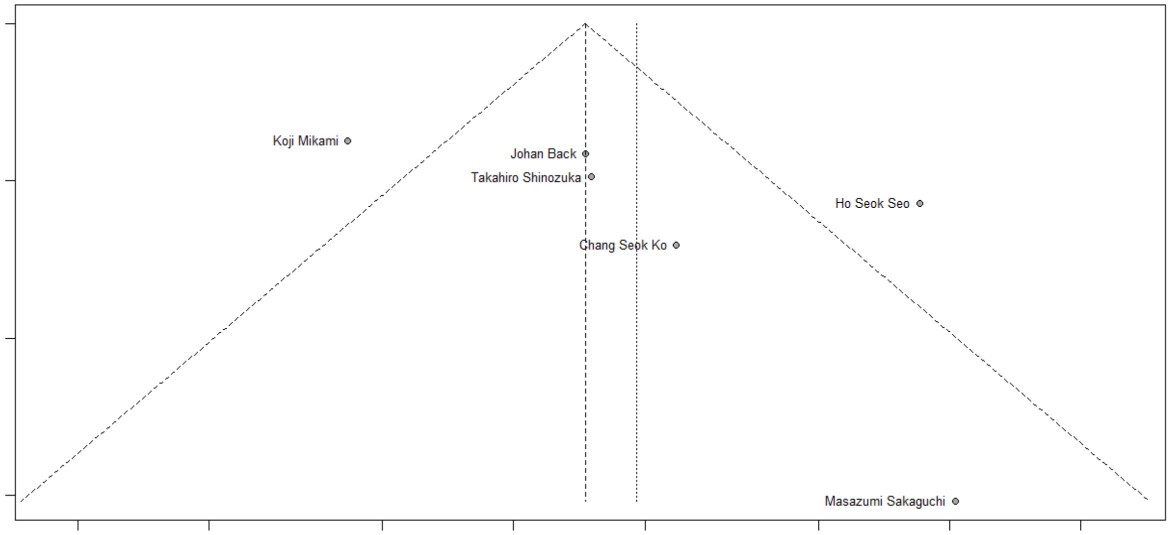

e. Age as a risk factor for OS

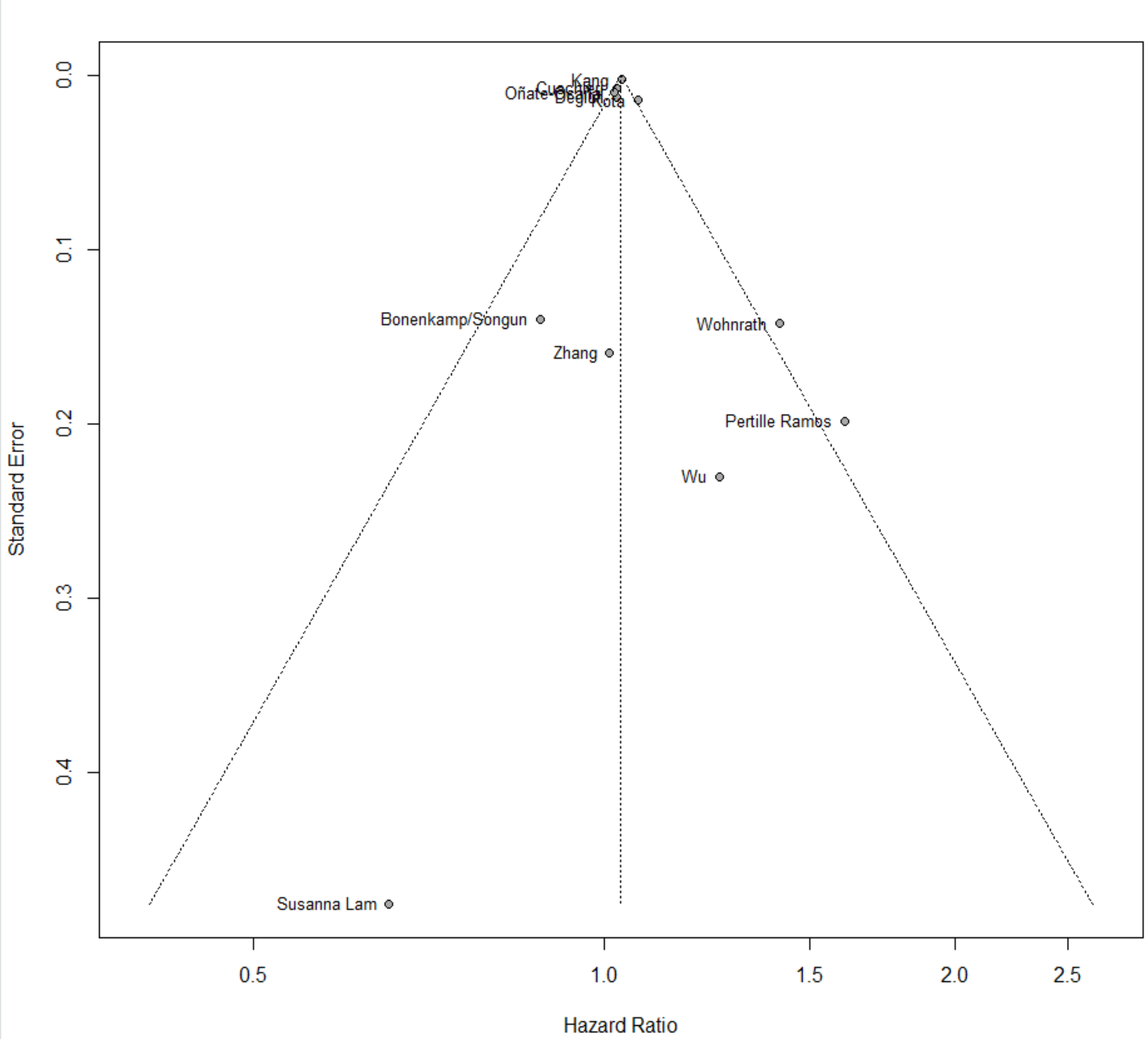

Kang
Cuachia
Oñate
Degli
Kota
Bonenkamp/Songun
Zhang
Wohnrath
Pertille Ramos
Wu
Susanna Lam
Standard Error
0.0
0.1
0.2
0.3
0.4
0.5
1.0
1.5
2.0
2.5
Hazard Ratio
