# Supplementary figures and images for: Lymph Node Dissection of Choice in Older Adult Patients with Gastric Cancer: A Systematic Review and Meta-Analysis
Source: J Clin Med. 2024 Dec 17;13(24):7678. doi: 10.3390/jcm13247678 (PMC11678213; doi:10.3390/jcm13247678)

#### S4. Subgroup analysis

##### a. Study design

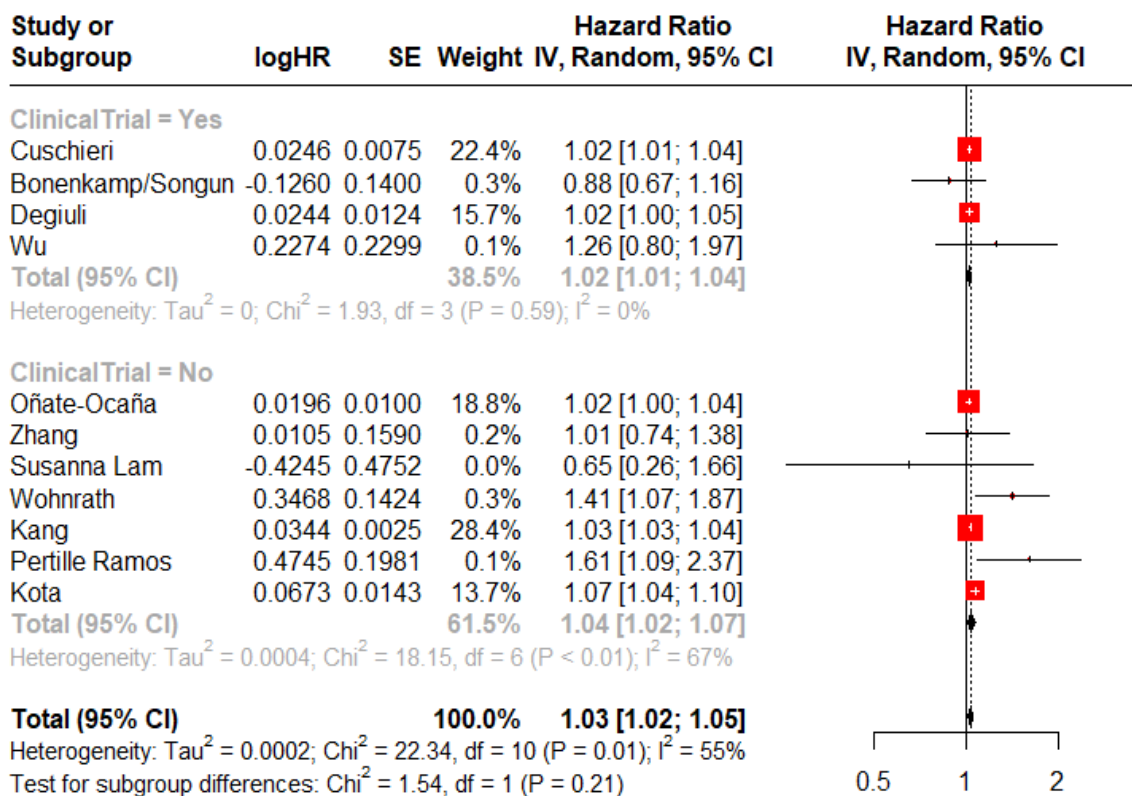

##### b. Publication year

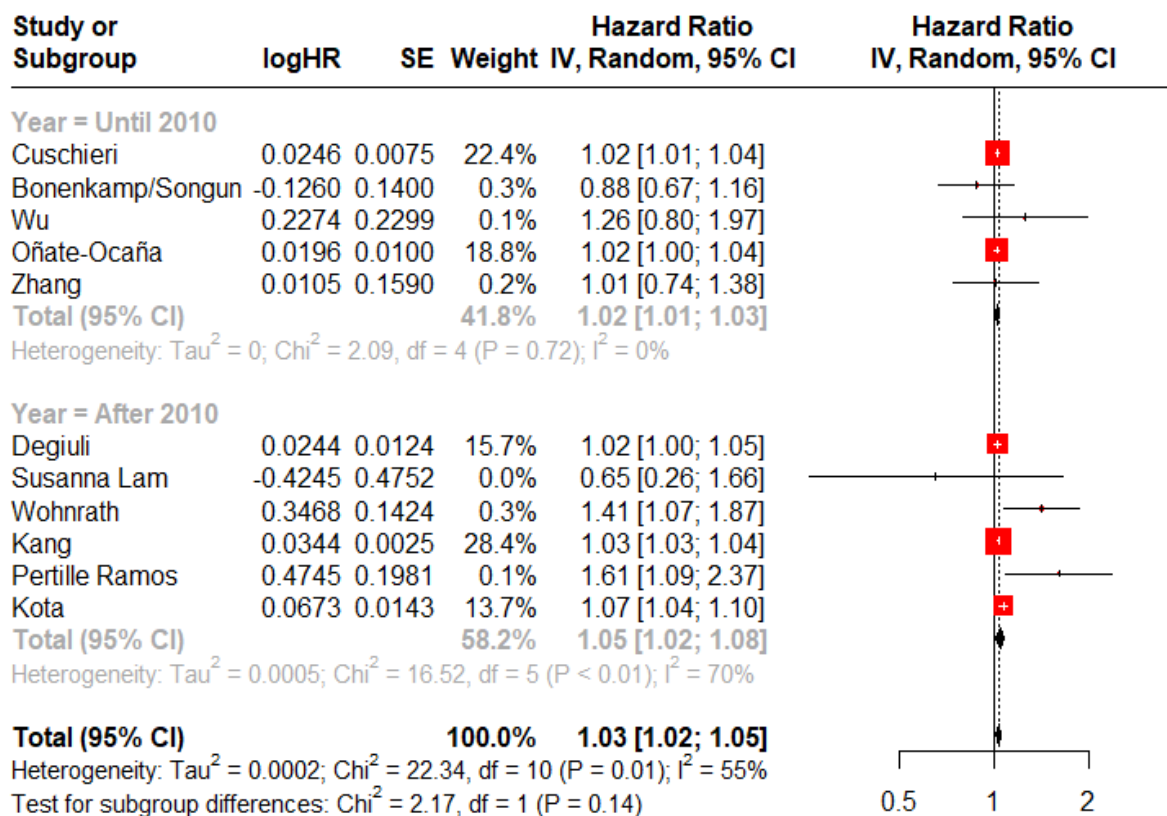

Supplement: Supplementary file 1 [file jcm-13-07678-s001.zip › S4. Subgroup analysis.pdf]
